# A 3′‐pre‐tRNA‐derived small RNA *tRF‐1‐Ser* regulated by 25(OH)D promotes proliferation and stemness by inhibiting the function of MBNL1 in breast cancer

**DOI:** 10.1002/ctm2.1681

**Published:** 2024-05-09

**Authors:** Xinyu Wan, Wenjie Shi, Lingjun Ma, Lexin Wang, Ran Zheng, Jinzhi He, Ye Wang, Xuan Li, Xiaoming Zha, Jue Wang, Lu Xu

**Affiliations:** ^1^ Department of Breast Disease The First Affiliated Hospital of Nanjing Medical University Nanjing China; ^2^ Department of Nutrition The First Affiliated Hospital of Nanjing Medical University Nanjing China

**Keywords:** 25‐hydroxyvitamin D, MBNL1, nutrition, RNA splicing, tRNA‐derived fragments

## Abstract

**Background:**

We explored the potential novel anticancer mechanisms of 25‐hydroxyvitamin D (25(OH)D), a vitamin D metabolite with antitumour effects in breast cancer. It is stable in serum and is used to assess vitamin D levels in clinical practice. Transfer RNA‐derived small RNAs are small noncoding RNAs that generate various distinct biological functions, but more research is needed on their role in breast cancer.

**Methods:**

Small RNA microarrays were used to explore the novel regulatory mechanism of 25(OH)D. High‐throughput RNA‐sequencing technology was used to detect transcriptome changes after 25(OH)D treatment and *tRF‐1‐Ser* knockdown. RNA pull‐down and high‐performance liquid chromatography–mass spectrometry/mass spectrometry were used to explore the proteins bound to *tRF‐1‐Ser*. In vitro and in vivo functional experiments were conducted to assess the influence of 25(OH)D and *tRF‐1‐Ser* on breast cancer. Semi‐quantitative PCR was performed to detect alternative splicing events. Western blot assay and qPCR were used to assess protein and mRNA expression.

**Results:**

The expression of *tRF‐1‐Ser* is negatively regulated by 25(OH)D. In our breast cancer (BRCA) clinical samples, we found that the expression of *tRF‐1‐Ser* was higher in cancer tissues than in paired normal tissues, and was significantly associated with tumour invasion. Moreover, *tRF‐1‐Ser* inhibits the function of MBNL1 by hindering its nuclear translocation. Functional experiments and transcriptome data revealed that the downregulation of *tRF‐1‐Ser* plays a vital role in the anticancer effect of 25(OH)D.

**Conclusions:**

In brief, our research revealed a novel anticancer mechanism of 25(OH)D, unveiled the vital function of *tRF‐1‐Ser* in BRCA progression, and suggested that *tRF‐1‐Ser* could emerge as a new therapeutic target for BRCA.

## Graphical abstract

1 25‐Hydroxyvitamin D decreases the expression of tRF‐1‐Ser.

2 tRF‐1‐Ser promotes proliferation and stemness of BRCA cells.

3 MBNL1 can be hindered from the nucleus by tRF‐1‐Ser, which contributes to the activation of JNK.

4 The suppression of tRF‐1‐Ser is crucial for the anticancer impact of 25‐hydroxyvitamin D.

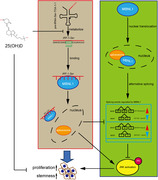

## 1 INTRODUCTION

Breast cancer is the predominant malignancy affecting women worldwide. Although advancements in modern medical science are gradually mitigating the therapeutic complexities associated with breast cancer, it still has the second highest mortality rate among carcinomas in women.^1^ Vitamin D, a prevalent nutritional element in the human body, has received attention from researchers because of its availability and broad spectrum of anticancer mechanisms. Many in vitro and in vivo experiments have demonstrated the robust anticancer effects of vitamin D in breast cancer, such as inhibiting tumour cell proliferation, promoting apoptosis, suppressing metastasis and invasion, and stimulating cancer cell differentiation.^2^, ^3^, ^4^ However, there is still limited clinical and epidemiological evidence regarding the anticancer effects of vitamin D. Emerging evidence has associated vitamin D deficiency with an unfavourable prognosis in breast cancer patients.^5^, ^6^, ^7^ However, a recent large‐scale randomised controlled clinical trial, VITAL, reported that the supplementation of vitamin D did not significantly reduce the incidence of cancer.^8^ Concurrently, no significant association has been found between serum 25‐hydroxyvitamin D (25(OH)D) levels and the pathological characteristics of breast cancers.^9^ Therefore, the mechanism of vitamin D effects requires further elucidation.

Vitamin D manifests in diverse metabolites, including 25(OH)D, 1,25‐dihydroxyvitamin D (1,25(OH)2D) and 1,24,25‐dihydroxyvitamin D (1,24,25(OH)3D). Among these, 25(OH)D, the most stable circulating metabolite, is a prevalent clinical indicator for assessing the overall vitamin D status in individuals. The biologically active form, 1,25(OH)2D, exerts its physiological effects by binding to the vitamin D receptor (VDR). It is catalysed from 25(OH)D by the enzyme 1α‐hydroxylase (CYP27B1), which is distributed in various tissues, mainly in the kidneys and in extrarenal tissues, such as the mammary and prostatic tissues.^10^ The highly bioactive 1,25(OH)2D can be converted to the biologically inactive 1,24,25(OH)3D. Consequently, CYP27B1, CYP24A1 and VDR collectively emerge as decisive factors governing the functional dynamics of vitamin D.

Currently, the predominant focus of research lies on 1,25(OH)2D, but recent investigations have also documented the anticancer effects of 25(OH)D in breast cancer.^11^, ^12^ Moreover, 25(OH)D is the most commonly employed indicator in clinical research to assess serum vitamin D levels. A recent meta‐analysis further consolidated the significant association between 25(OH)D deficiency and an adverse prognosis in female breast cancer patients.^5^ Moreover, CYP27B1 is also expressed in breast tissue, where 25(OH)D can be converted into 1,25(OH)2D via local synthesis. Local synthesis differs from renal synthesis in that it is not regulated by calciotropic hormones, but rather, it is dependent on the serum concentration of 25(OH)D.^10^ Elevated local concentrations of 25(OH)D also contribute to increased generation of 1,25(OH)2D within breast tumour tissues. This has been applied in clinical research, wherein high‐dose oral supplementation of vitamin D was found to elevate serum 25(OH)D levels while avoiding potential drug toxicities such as hypercalcaemia. In this study, we focused on investigating 25(OH)D, elucidating its mechanistic role in breast cancer.

Transfer RNA‐derived small RNAs (tsRNAs) are a novel class of small noncoding RNAs broadly categorised into two main groups based on their nucleotide length and origin within tRNA: tRNA halves (tiRNAs) and tRNA‐derived fragments (tRFs). Among them, tiRNA arises from specific cleavage sites within the anticodon loop through angiogenin (ANG) activity, and it is typically generated in response to various stress conditions.^13^, ^14^ Conversely, tRFs are a class of small RNAs with lengths ranging from 14 to 32 nucleotides that originate from either mature tRNA or tRNA precursors. Depending on their mapping locations to tRNA or tRNA precursors, this can be further subdivided into four categories: tRF‐5, tRF‐3, i‐tRF and tRF‐1.^15^ Several studies have elucidated the impact of tiRNAs and tRFs on cancer progression. For instance, under hypoxic conditions, breast cancer cells with reduced invasive and metastatic capabilities generate a set of tRFs sharing a common motif. These tRFs competitively bind to the RNA‐binding protein (RBP) YBX1, displacing various pro‐oncogenic transcripts that would otherwise bind to YBX1. This process destabilises pro‐oncogenic transcripts, subsequently inhibiting the invasion and metastasis of tumour cells.^16^ In addition, tRF‐1001, a tRF‐1 derived from the 3′ end of pre‐tRNA‐Ser‐TGA by ELAC2 cleavage, is highly expressed in various tumour cells based on sequencing data, with lower expression observed in normal tissue. Silencing tRF‐1001 using siRNA significantly inhibits the capacity of prostate cancer cells.^17^ Therefore, tsRNAs play a pivotal role in the development of carcinoma, warranting further in‐depth exploration of their mechanistic implications.

Transcriptome data from Rabbani and coworkers revealed the biological processes influenced by 25(OH)D, including the tRNA catabolic process. Similarly, our gene set enrichment analysis (GSEA) results revealed that 25(OH)D impacts the metabolic processes and modification of tRNA, which has sparked significant interest. Subsequent small RNA‐sequencing (RNA‐seq) data in our study identified several tsRNAs regulated by 25(OH)D, wherein *tRF‐1‐Ser* derived from pre‐tRNA‐Ser‐TGA showed the most notable influence from 25(OH)D. *tRF‐1‐Ser* also can be named as tDR‐T1:T20‐Ser‐TGA‐1‐1 according to tDRnamer,^18^ a latest naming rule of tRNA‐derived RNAs based on molecular origin. Interesting, it also has a striking similarity to tRF‐1001 mentioned above. Thus, our investigation delves into the intricate interplay between *tRF‐1‐Ser* and 25(OH)D.

## 2 MATERIALS AND METHODS

### 2.1 Clinical samples

All BRCA and paired normal tissues were taken from patients who had surgeries at Jiangsu Province Hospital between 2021 and 2022. All tissues were preserved in RNALater preserving liquid (Beyotime) at −80°C. All patients were diagnosed via precise histopathological examination, and the patient's clinical characteristics were obtained from electronic medical records. All specimens were collected with consent of the patient. The Ethics Committee of Jiangsu Province Hospital approved this study.

### 2.2 Cell lines

The human BRCA cell lines MDA‐MB‐453, MDA‐MB‐415, MDA‐MB‐468, MDA‐MB‐231, BT474, SKBR3 and MCF‐7, and immortalised human breast cell line MCF‐10a were kindly gifted by Dr. Ziyi Fu. All BRCA cell lines were cultured in Dulbecco's modified Eagle medium (Gibco) supplemented with 10% foetal calf serum (Lonsera) and 1% penicillin–streptomycin (Servicebio). The cells were cultured at a temperature of 37°C and in an environment with 5% CO_2_.

### 2.3 Animal experiments

Animal experiments were performed according to the guidelines of the Jiangsu Animal Experimental Center of Medical and Pharmaceutical Research and were approved by the Institutional Animal Care and Use Committee of Nanjing Medical University. Tumourigenesis assays were performed on 5‐week‐old BALB/c nude mice, which were divided into four groups in random (*n* = 5 each). In the *tRF‐1‐Ser* overexpression assay, MDA‐MB‐468 cells were injected subcutaneously into the left upper back of the mice, followed by a local injection of ago*tRF‐1‐Ser* (RIBOBIO) around the subcutaneous tumour (10 nmol per mouse) after 1 week. Phosphate‐Buffered Saline (PBS) was used as the control. In the *tRF‐1‐Ser* knockdown assay, MDA‐MB‐468 cells stably transfected with sh*tRF‐1‐Ser* or shNC (negative control) were used. The tumours' major (*a*) and minor (*b*) axes were measured using a digital calliper every 3 days, and the formula *V* = *ab* ^2^/2 was used to calculate the tumour volume. After 4 weeks, the tumours were dissected and weighed.

### 2.4 Access and analysis of public data

The RNA‐seq data of BRCA were accessed from The Cancer Genome Atlas (TCGA) (https://www.cancer.gov/tcga) with the R package ‘easyTCGA’. Transcripts per kilobase per million mapped reads (TPM) were used to quantify gene expression. A total of 1231 cases were used to analyse the enrichment pathway of MBNL1.

### 2.5 Small RNA microarray analysis

MDA‐MB‐453 cells underwent growth in two different environments. The experimental group (EG) was exposed to 100 nM 25(OH)D, whereas the control group (CG) was cultured in .1% alcohol for 5 days. To ensure accuracy, each group was repeated thrice. For each sample, 100 ng of total RNA was then dephosphorylated to form the 3‐OH end, followed by enzymatic labelling with Cy3. The RNA was labelled and hybridised onto an Arraystar Human small RNA Microarray (8 × 15K, Arraystar), which was then scanned with an Agilent Scanner G2505C. Shanghai Aksomics performed all the steps aside from sample preparation. Data from the small RNA microarray can be accessed in the GEO database with access number GSE250048.

### 2.6 RNA extraction and Reverse transcription‐quantitative PCR (qRT‐PCR)

Total RNA was isolated using TRIzol reagent (Takara). Total RNA (1 μg) was used to synthesise first‐strand cDNA using Hifair III 1st Strand cDNA Synthesis Kit (Yeasen). Stem‐loop RT primers were applied for reverse transcription of tRF and tiRNA, while qRT‐PCR was conducted using Hieff qPCR SYBR Green Master Mix (Yeasen) with QuantStudio 7 (Thermo Fisher). The 2^−ΔΔCt^ method was applied to calculate fold changes in the target genes. The sequences of qRT‐RCR primers can be found in Table S1.

### 2.7 Absolute quantification by PCR

The standard tRF was synthesised from Guangzhou RIBOBIO. The gradient concentration of standard RNA was set from 10^5^ to 1 fM, and first‐strand cDNA was synthesised. The standard curve was acquired by qRT‐PCR by applying standard cDNA. The relationship between *tRF‐1‐Ser* CT values and *tRF‐1‐Ser* concentration was calculated with linear model.

### 2.8 Semi‐quantitative PCR

Total RNA (1 μg) isolated from BRCA cell lines was reverse transcribed into cDNA. HiScript II One‐Step RT‐PCR Kit (Vazyme) was used to perform PCR. The PCR products were separated using electrophoresis technique on 3% agarose gels with NA‐Red (Beyotime) to obtain the desired results. Images were acquired using ChemiDocXRS (BIO‐RAD). ImageJ was used to quantify long and short isoform signals. The sequences of semi‐quantitative PCR primers are listed in Table S1.

### 2.9 Transfection of *tRF‐1‐Ser* mimic, small interfering RNA and plasmids

The *tRF‐1‐Ser* overexpression model was constructed by transfecting synthetic *tRF‐1‐Ser* mimic, while the scrambled RNA was transfected as a negative control. The *tRF‐1‐Ser* knockdown model was accomplished by transfecting sh*tRF‐1‐Ser* plasmid packed into lentivirus, and the negative control lentivirus was transfected as the control. Small interfering RNAs (siRNAs) against MBNL1 were used to construct the MBNL1 knockdown model. The MBNL1 overexpression plasmid was purchased from GeneChem. All mimic, siRNAs and lentiviruses were ordered from RIBOBIO. Lipofectamine 3000 reagent (Invitrogen) was used for cell transfection. Lentivirus transfection was performed in accordance with the manufacturer's protocol.

### 2.10 Cell counting kit 8

BRCA cells were plated into 96‐well plates at a density of 3000 cells. The cell culture medium was removed and fresh culture medium containing 100 nM of 25(OH)D or RNA‐Lipo3000 mixture was added. After 24, 48, 72, 96 and 120 h after the cells were plated into 96‐well plates, cell counting kit 8 (CCK8) reagent was applied to each well and cultured at 37°C for 1 h. Pro‐11 Multiskan FC (Thermo Fisher) was used to examine each well's optional density at 450 nm. Each assay was duplicated thrice.

### 2.11 Cell‐light 5‐ethynyl‐2'‐deoxyuridine (EDU) Apollo in vitro kit (EDU assay)

BRCA cells were plated into 96‐well plates at a density of 20 000 cells. After 24 h, the culture medium was removed and fresh culture medium containing 100 nM of 25(OH)D or RNA‐Lipo3000 mixture was added. After 96 h after the cells were plated, the EDU assay was performed following the guidelines provided by the manufacturer. The fluorescences of EDU and 4′,6‐diamidino‐2‐phenylindole (DAPI) were collected using THUNDER DMi8 (LECIA). The cell count was determined using ImageJ.

### 2.12 Cell cycle staining kit

BRCA cells were harvested from a six‐well plate and fixed with 75% alcohol at −20°C for at least 24 h. The staining steps were performed following the Cell Cycle Staining Kit protocol (MULTI SCIENCES). After hydrating the cells, 1 mL of DNA staining solution was added to each sample and samples were incubated at 20°C for 30 min. The cell cycle data were collected by Cytoflex (Beckman).

### 2.13 Flow cytometry for breast cancer stem cell detection

Flow cytometry was applied to detect the proportion of breast cancer stem cells (BCSCs). BRCA cells were dissociated from a six‐well plate and suspended in flow cytometry staining buffer (Thermo Fisher). Cells were labelled with conjugated antihuman CD44‐Phycoerythrin (PE), CD24‐Fluorescein Isothiocyanate (FITC) or EpCAM‐PE (Thermo Fisher), incubated for at least 30 min on ice in dark, then washed twice with flow cytometry washing buffer. Finally, PE or FITC was detected using Cytoflex from Beckman.

### 2.14 Tumour‐sphere formation assay

BRCA cells were pretreated in the six‐well plates for 3 days and then subjected to the tumour‐sphere formation assay. Treated breast cancer cells were cultured (10 000 cells/well) in ultralow attachment six‐well plates (Corning) with MammoCultTM Basal Medium (STEMCELL Technologies). After 7 days of seeding cells, the tumour spheres were counted and photographed using a light microscope (OLYMPUS‐CKX53).

### 2.15 mRNA sequencing

Biological replicates were set to ensure the repeatability of mRNA sequencing (mRNA‐seq). MDA‐MB‐453 was cultured in 100 nM of 25(OH)D for the EG or .1% alcohol for the CG. MDA‐MB‐453 transfected with sh*tRF‐1‐Ser* or shNC by lentivirus was named lv‐shtRF or lv‐con. DNA digestion was conducted by DNaseI after extracting RNA. Total RNA (2 μg) was used to construct an RNA‐seq library with the KCTM Stranded mRNA Library Prep Kit for Illumina (Wuhan SeqHealth). The PCR products were analysed with a PE150 model using a Novaseq 6000 sequencer (Illumina).

The mRNA‐seq experiment and data analysis were accomplished by SeqHealth Technology. GSEA (version 4.3.2) was used to analyse the enrichment pathway. Alternative splicing events were examined using rMATS (version 3.2.5). Different gene expressions between groups were identified using the edgeR package (version 3.12.1). The RNA‐seq data were uploaded to the GEO database, with the accession numbers GSE247667 and GSE249937.

### 2.16 RNA pull‐down assay

The biotin‐labelled tRF‐1‐Ser was purchased from RIBOBIO. The Magnetic RNA–Protein Pull‐Down Kit (Thermo Fisher) was used according to the manufacturer's instructions. First, the magnetic beads were mixed with 100 pmol of biotin‐labelled RNA and incubated at 20°C for 30 min. After that, the RNA–protein mixture was added to the beads and incubated at 4°C for 60 min. The immunoprecipitation product was separated by sodium dodecyl sulfate‐polyacrylamide gel electrophoresis (SDS–PAGE and identified using the Fast Silver Stain Kit (Beyotime). Finally, the RNA pull‐down products were analysed through high‐performance liquid chromatography–mass spectrometry/mass spectrometry (HPLC–MS/MS) performed by Nanjing Jiangbei New Area Biopharmaceutical Public Service Platform.

### 2.17 Western blotting

Total protein was extracted using RIPA lysis buffer (Beyotime). Cytoplasmic and nuclear proteins were separated with the Nuclear and Cytoplasmic Protein Extraction Kit (Beyotime). BCA Protein Assay Kit (Beyotime) was utilised to detect the concentration of protein. The total protein (20 μg) was separated by 7.5% or 10% SDS–PAGE (Epizyme) and then transferred onto polyvinylidene fluoride (PVDF) membranes (Millipore). QuickBlock Blocking Buffer for Western Blot (Beyotime) was applied in blocking PVDF membranes. Table S1 lists specific information about antibodies used in the Western blotting assay.

### 2.18 RBP immunoprecipitation

RNA immunoprecipitation (RIP) assay was performed using Magna RIP RNA‐Binding Protein Immunoprecipitation Kit (Sigma–Aldrich) according to the manufacturer's protocol with MBNL1 antibody (Thermo Fisher). After incubating magnetic beads with 5 μg of MBNL1 antibody at 20°C for 30 min, the beads were incubated with a protein–RNA mixture at 4°C overnight. The immunoprecipitation RNA was extracted using TRIzol reagent (Takara). Target gene expression in the immunoprecipitation product was quantified using qRT‐PCR.

### 2.19 Fluorescence in situ hybridisation and immunofluorescence assay

Fluorescence in situ hybridisation (FISH) and immunofluorescence (IF) assays were conducted to verify colocalisation and detect the subcellular distribution of *tRF‐1‐Ser* and MBNL1. FISH assay was performed using Ribo FISH (RIBOBIO) with a *tRF‐1‐Ser* FISH probe synthesised by RIBOBIO following instructions provided the manufacturer. For IF assay, cells were fixed with 4% paraformaldehyde fix solution at 20°C−25°C for 20 min and permeated with Immunostaining Permeabilisation Buffer with Triton X‐100 (Beyotime) for 5 min. Immunol Staining Blocking Buffer (Beyotime) was used to block the nonspecific binding reactions. Primary antibodies against MBNL1 (1:100, Thermo Fisher) and secondary antibodies coupling FITC (1:50, Jackson) were used for IF. The cell nucleus was stained with DAPI (Beyotime). The Cy3, FITC and DAPI signals were detected via Stellaris STED laser scanning confocal microscopy (LEICA). ImageJ was applied to analyse the colocalisation.

### 2.20 Haematoxylin–eosin staining, immunochemistry and IF of tissues

After tumour dissection, the tissues were fixed with 4% Paraformaldehyde Fix Solution (Beyotime) at 4°C for 6 h. Haematoxylin–eosin (HE) staining, immunohistochemistry (IHC) and IF analyses of tissues were conducted by Servicebio.

### 2.21 Electrophoretic mobility shift assay

Purified MBNL1 protein (CUSABIO), cytoplasmic or nuclear protein separated from MDA‐MB‐453, was incubated with *tRF‐1‐Ser* biotin‐labelled (RIBOBIO), *tRF‐1‐Ser* biotin‐unlabelled (RIBOBIO), MBNL1 antibody (Thermo Fisher) or *tRF‐1‐Ser*‐UGCU‐mutation biotin‐labelled (RIBOBIO) in REMSA Binding Buffer (Beyotime) at 4°C for 10 min. The RNA–protein complex was separated with a 6% Tris‐Borate‐EDTA buffer (TBE) PAGE gel in ice box, then transferred to a nylon membrane (Beyotime). The membrane was then incubated with streptavidin–horseradish peroxidase (HRP) conjugate at 20°C for at least 20 min. The signal of the RNA–protein complex was detected using ChemiDocXRS (BIO‐RAD).

### 2.22 Northern blot

Biotin Northern Blot Kit (Beyotime) was used to conduct a Northern blot (NB). ssDNA NB biotin‐labelled probe synthesised by RIBOBIO was applied to detect the abundance of *tRF‐1‐Ser* and *U6*. The total RNA was denatured at 65°C for 15 min and then separated by 15% urea‒PAGE. Urea‒PAGE was stained with NA‐red (Beyotime) to observe the separation condition of total RNA. The total RNA was then transferred onto a nylon membrane (Beyotime) and prehybridised with Quick Hybridisation Solution at 42°C for 2 h. The nylon membrane was hybridised with *tRF‐1‐Ser* or U6 NB biotin‐labelled probe at 42°C for 12 h. After washing, the membrane was incubated with streptavidin–HRP conjugate solution and the signal was detected using ChemiDocXRS (BIO‐RAD).

### 2.23 Statistical analysis

The results are presented as the mean ± standard deviation or individual plots from at least three independent experiments. To determine the significance between two groups, appropriate statistical tests such as Student's *t*‐test or analysis of variance were conducted. Paired *t*‐test was used to compare the expression of *tRF‐1‐Ser* in 62 BRCA tissues and paired normal breast tissue. Chi‐square test was used to examine the correlation between the expression of *tRF‐1‐Ser* and the clinical features of patients. GraphPad Prism 9 was utilised to perform statistical analysis, with *p*‐value <.05 considered statistically significant (^*^ *p* < .05, ^**^ *p* < .01, ^***^ *p* < .001).

## 3 RESULTS

### 3.1 25(OH)D suppresses the proliferation of breast cancer cells and arrests the cell cycle in G1

**FIGURE 1:**
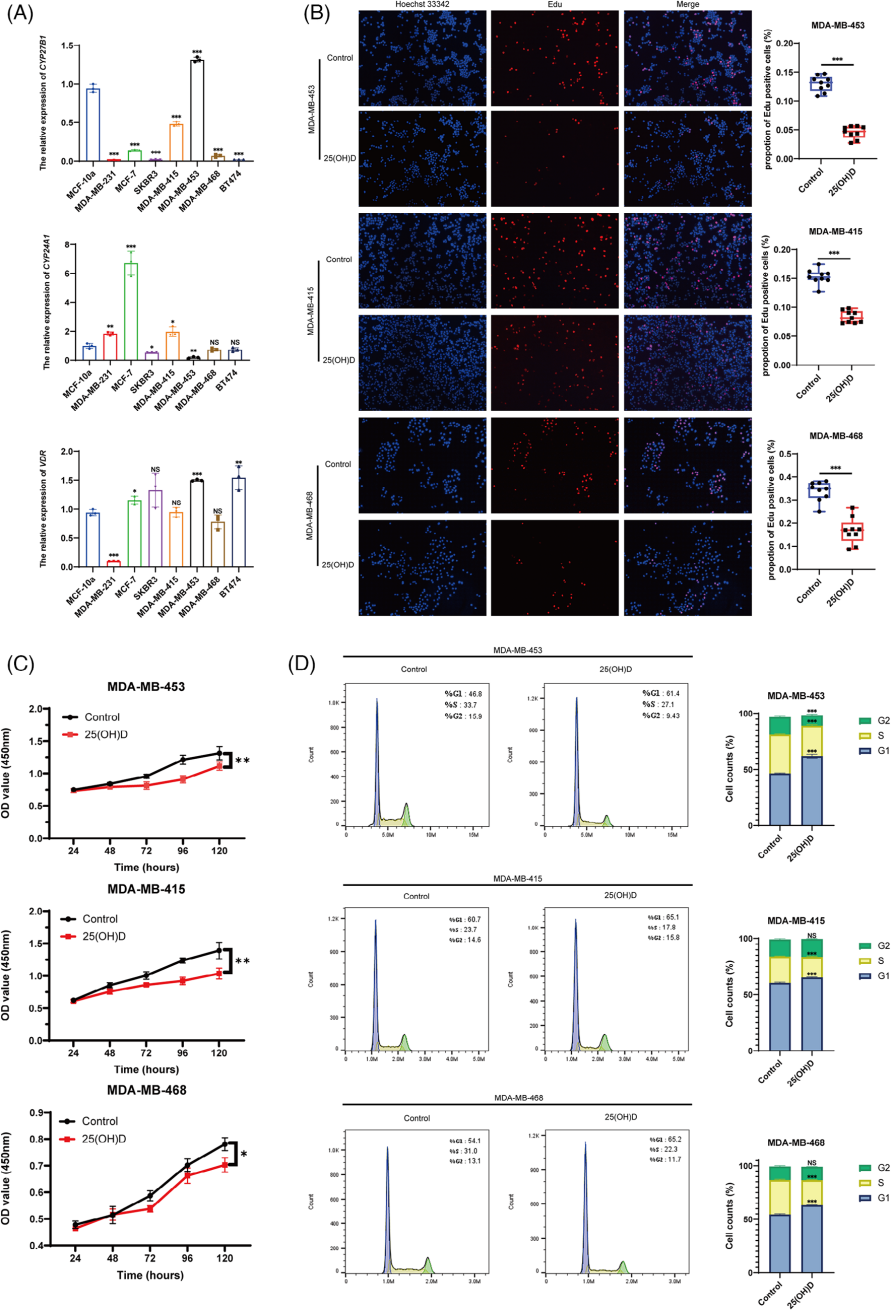
25‐Hydroxyvitamin D (25(OH)D) suppresses the proliferation of breast cancer cells and arrests cell cycles in G1. (A) The relative expression of CYP27B1, CYP24A1 and vitamin D receptor (VDR) in BRCA cell lines and MCF‐10a detected by qPCR. (B) EDU assay was performed to detect the influence of 25(OH)D on the proliferation of BRCA cell lines. ImageJ was applied to count cell numbers and an unpaired *t*‐test was carried out to calculate the significance of two groups. (C) Cell counting kit 8 (CCK8) was carried out to detect the growth curve of BRCA cells cultured with 100 nM 25(OH)D. Two‐way analysis of variance (ANOVA) was performed to calculate the significance of two groups. Data are shown as mean ± standard deviation (SD). (D) The results of flow cytometry showed the percentage of cells in the G1, S and G2 phases, presented in a bar graph. Data are shown as mean ± SD. Unpaired *t*‐test was carried out to calculate the statistical significance compared to the control. ^*^ *p* < .05, ^**^ *p* < .01, ^***^ *p* < .001.

Considering the crucial regulatory roles of CYP27B1, CYP24A1 and VDR in the vitamin D pathway, we initially assessed their relative expression levels in various BRCA cell lines compared to MCF‐10a, an immortalised normal breast epithelial cell line, to identify cells that might be responsive to 25(OH)D (Figure 1A). CYP27B1 and VDR expression were significantly elevated in MDA‐MB‐453, MDA‐MB‐415 and MDA‐MB‐468 cells. In contrast, the expression of CYP24A1 was relatively diminished. For subsequent research, we identified three cell lines and designated MDA‐MB‐231 as the control. Our selection of MDA‐MB‐231 was based on its gene expression pattern of CYP27B1, CYP24A1 and VDR, which is in direct contrast to the three aforementioned cell lines. Physiological concentrations of 25(OH)D (100 nM) have been shown to inhibit the proliferative capacity of tumour cells in vitro.^12^, ^19^ To examine the effects of 25(OH)D on breast cancer cells, we cultured breast cancer cells in a medium supplemented with 100 nM 25(OH)D or a corresponding solvent, then evaluated their proliferative capacity via CCK8 and EDU experiments. Adding 25(OH)D to the culture medium reduced the proliferative capacity of all three cell lines at varying degrees. MDA‐MB‐453 showed the most significant reduction in proliferative capacity (Figure 1B,C). As anticipated, 25(OH)D did not affect the proliferative capacity of MDA‐MB‐231 cells (Figure S1B,C).

Flow cytometry was applied to detect the cell cycle, and we found that 25(OH)D significantly inhibited the transition of the three tumour cell lines from the G1 to S phase (Figure 1D). Corresponding to the observed impact on cell proliferation, the cell cycle arrest effect of 25(OH)D was particularly pronounced in MDA‐MB‐453 cells. Microscopic examination revealed marked alterations in the shape of MDA‐MB‐453 cells after 25(OH)D treatment, transitioning from an elliptical to a flattened state (Figure S1D), which is same with the reported effects induced by 1,25(OH)_2_D.^20^ Therefore, physiological concentrations of 25(OH)D exert an inhibitory effect on the proliferative capacity of breast cancer cells. In addition, this inhibitory effect may be associated with the autocrine and paracrine secretion capabilities of tumour cells.

### 3.2 25(OH)D can regulate the production of tsRNAs by affecting tRNA metabolism

**FIGURE 2:**
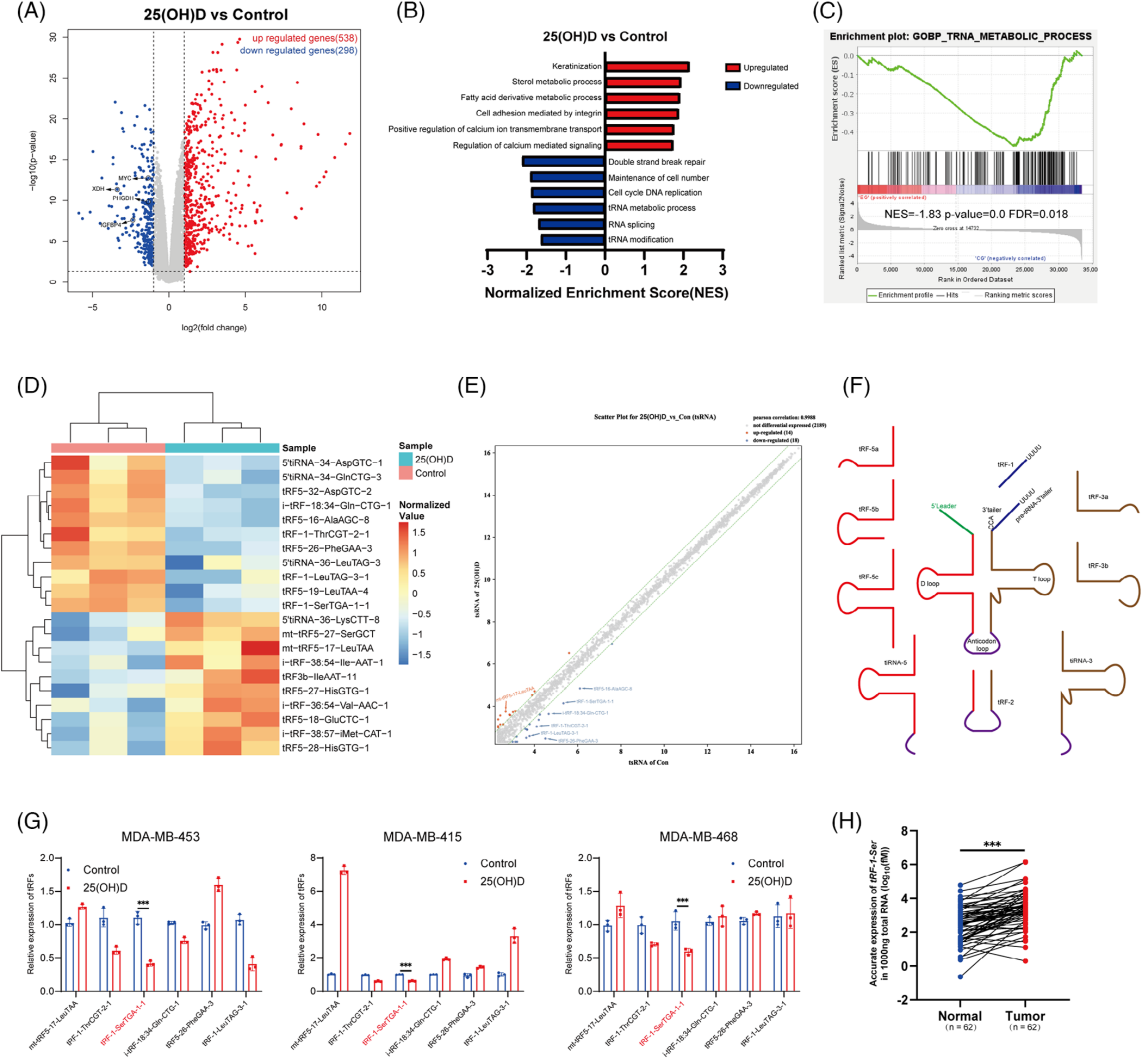
25‐Hydroxyvitamin D (25(OH)D) can regulate the production of transfer RNA‐derived small RNAs (tsRNAs) by affecting the tRNA metabolism process. (A) Genes were shown with the volcano plot with significant differential genes marked red (upregulation) or blue (downregulation). A significant difference was defined as |log2(fold change)| > 1 and *p*‐value < .05. (B) Gene set enrichment analysis (GSEA) was performed with the gene set of GOBP and part of the analysis was shown with bar graphs. (C) The output of ‘GOBP_TRNA_METABOLIC_PROCESS’ analysed with GSEA is shown. (D) Hierarchical clustering heatmap of differentially expressed tsRNAs. (E) A scatter plot displays the normalised intensities of tsRNAs in two compared groups on the *X* and *Y* axes. (F) The location of tsRNAs mapped to tRNA. (G) The change in the relative expression of tRNA‐derived fragments (tRFs) in BRCA cells cultured with 25(OH)D was detected by qPCR. U6 was applied as a control of qPCR. An unpaired *t*‐test was applied to calculate significance. Data are shown as mean ± standard deviation (SD). (H) The accurate expression of tRF‐1‐Ser in BRCA tissues and paired normal tissues was detected by qPCR. Paired *t*‐test was carried out to calculate the statistical significance. ^*^ *p* < .05, ^**^ *p* < .01, ^***^ *p* < .001.

To understand how 25(OH)D affects breast cancer cells, we examined MDA‐MB‐453 cells using next‐generation transcriptome sequencing. Cells were cultured with 100 nM 25(OH)D, while the CG was cultured in a solvent. Gene expression analysis revealed 538 and 296 upregulated and downregulated genes, respectively (Figure 2A). Subsequently, GSEA using Gene Ontology (GO) biological process gene sets was employed to explore the impact of 25(OH)D on breast cancer biological processes (Figure 2B).

Enrichment analysis of MDA‐MB‐453 cells treated with 25(OH)D revealed significant upregulation of biological processes such as cell adhesion mediated by integrin and keratinisation. This is in line with the observed changes in cell morphology (Figure S1D). Conversely, downregulated biological processes included ‘maintenance of cell number’ and ‘cell cycle DNA replication’. These terms were validated through phenotypic experiments. Unexpected and less‐documented terms, such as ‘tRNA metabolism’ and ‘tRNA modification’, were also identified among the downregulated biological processes (Figure 2C). This is an interesting finding because recent studies have indicated that tRFs from tRNA degradation metabolism are significant and play key roles in biological processes such as cell cycle regulation and apoptosis induction.^21^ Based on the GSEA, we selected three ribonucleases involved in tRNA metabolism (ANG, TSEN2, ELAC2) to validate the changes in protein levels under the influence of 25(OH)D (Figure S2C). All three ribonucleases were variably reduced, with ELAC2 showing less pronounced changes, which is consistent with our RNA‐seq findings. This strongly suggests that 25(OH)D may influence tRNA metabolism.

We used small RNA microarrays to detect changes in the expression of tRFs in MDA‐MB‐453 cells after 25(OH)D exposure. We identified the differentially expressed tRFs by calculating the fold changes and *p*‐values for each tRF. Those with |fold change| ≥1.5 and *p*‐value <.05 were considered to display differential expression. The differentially expressed tRFs were visualised using a heatmap (Figure 2D), which showed that 10 and 11 tRFs were upregulated and downregulated, respectively. We also averaged the normalised expression levels of tRFs in both groups and plotted a scatter plot (Figure 2E), marking tRFs with |fold change| ≥2. Among them, tRF‐1 and tRF‐5 showed the most significant expression differences. The tsRNA nomenclature was based on its size and initiation site (Figure 2F).

Considering the potential bias introduced by using a single BRCA cell line, we further validated the expression changes of tsRNAs in three breast cancer cell lines cultured with 25(OH)D using qPCR (Figure 2G). We focused on tsRNAs with |fold change| ≥2, which revealed that tRF‐1 was the major downregulated type in breast cancer cells cultured with 25(OH)D. Among the downregulated tRF‐1, *tRF‐1‐SerTGA‐1‐1* (also named tDR‐T1:T20‐Ser‐TGA‐1‐1) exhibited the most significant decrease. The NB assay also indicated that tRF‐1‐Ser was downregulated by 25(OH)D (Figure S2I). Moreover, we collected clinical samples from 62 patients with breast cancer and adjacent tissues, from which the total RNA was extracted, revealing that *tRF‐1‐Ser* expression was often elevated in tumour tissues (Figure 2H). Based on clinical data, patients were categorised into high‐ and low‐expression groups based on *tRF‐1‐Ser* expression. Chi‐square tests were used to explored the correlations between *tRF‐1‐Ser* expression and clinical characteristics, which revealed that *tRF‐1‐Ser* expression was significantly correlated with tumour invasion and TNM stages (Table 1).

**TABLE 1:** Correlation between tRF‐1‐Ser and pathological features in 62 BRCA patients.

		tRF‐1‐Ser expression		
Characteristics	Numbers	Low	High	*p*‐Value
Total	62	30	32	
Age (years)				.3212
≤55	27	15	12	
>55	35	15	20	
Differentiation				.6221
I‒II	33	15	18	
III	29	15	14	
ki‐67				.3117
≤20	16	6	10	
>20	46	24	22	
Tumour invasion				.0019^**^
DCIS‒T1	27	20	7	
T2‒T3	35	10	25	
Lymph nodes metastasis				.302
N0	39	21	18	
N1‒N3	23	9	14	
TNM stage				.0014^**^
I stage	17	14	3	
II stage‒III stage	45	16	29	
Serum 25(OH)D level				.418
<50	56	26	30	
≥50	6	4	2	

*Note*: Chi‐square test was used to examine the correlation between the expression of tRF‐1‐Ser and the clinical features of patients.

Abbreviation: 25(OH)D, 25‐hydroxyvitamin D.

^**^ *p* < .01.

### 3.3 *tRF‐1‐Ser* promotes the proliferation of BRCA cells

**FIGURE 3:**
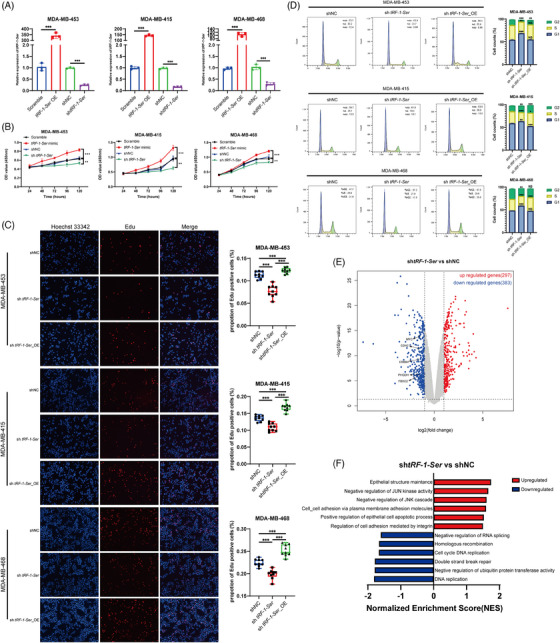
tRF‐1‐Ser can promote the proliferation of BRCA cells. (A) The efficiencies of knockdown and overexpression of tRF‐1‐Ser were verified by qPCR. U6 was applied as a control of qPCR. (B) Cell counting kit 8 (CCK8) was performed to detect the growth curve of BRCA cells treated with the overexpression or knockdown of tRF‐1‐Ser mimic. (C) EDU assay was used to detect the influence of tRF‐1‐Ser on the proliferation of BRCA cell lines. shtRF‐1‐Ser_OE refers that tRF‐1‐Ser was reintroduced to BRCA cells stably transfected with shtRF‐1‐Ser. (D) The effect of tRF‐1‐Ser on the cell cycle was detected by flow cytometry. Unpaired *t*‐test was carried out to calculate the statistical significance compared to shNC. (E) Genes were shown with the volcano plot with significant differential genes marked red (upregulation) or blue (downregulation). (F) Gene set enrichment analysis (GSEA) was performed with the gene set of GOBP and part of the analysis was shown with bar graphs. Data are shown as mean ± standard deviation (SD). ^*^ *p* < .05, ^**^ *p* < .01, ^***^ *p* < .001.

Considering the regulatory influence of 25(OH)D on *tRF‐1‐Ser*, we hypothesised that *tRF‐1‐Ser* is involved in the anticancer effects mediated by 25(OH)D. Therefore, we synthesised both *tRF‐1‐Ser* mimic and its corresponding shRNA, with the knockdown efficiency of shRNA previously reported in prostate cancer cells.^17^ Using cell lines responsive to 25(OH)D, we established models of *tRF‐1‐Ser* overexpression and knockdown, using qPCR to validate its efficiency (Figure 3A). Subsequently, we constructed stable cell lines with *tRF‐1‐Ser* knockdown via lentiviral infection. Cell growth curves were generated using CCK8 experiments, revealing that *tRF‐1‐Ser* knockdown significantly inhibited tumour cell growth, while *tRF‐1‐Ser* overexpression resulted in enhanced proliferative capacity in all three breast cancer cell lines (Figure 3B). EDU experiments confirmed that *tRF‐1‐Ser* knockdown weakened the proliferative ability of breast cancer cells, whereas proliferation was restored and even enhanced upon *tRF‐1‐Ser* reintroduction (Figure 3C). On cell cycle analysis, *tRF‐1‐Ser* knockdown led to cell cycle arrest in the G1 phase, whereas *tRF‐1‐Ser* reintroduction accelerated its transition from the G1 to the S phase (Figure 3D).

To explore the molecular mechanisms of *tRF‐1‐Ser* in breast cancer, we performed mRNA‐seq on stable cells with *tRF‐1‐Ser* knockdown and their controls. Differential gene expression analysis revealed 297 and 383 upregulated and downregulated genes, respectively, as depicted in the volcano plot (Figure 3E). GSEA revealed that JNK cascade activity and c‐JUN kinase were inhibited upon *tRF‐1‐Ser* knockdown (Figure 3F). Consistent with our previous observations, cell cycle DNA replication was also suppressed. In the enriched terms, the addition of 25(OH)D and *tRF‐1‐Ser* knockdown regulated the RNA splicing biological processes, suggesting that *tRF‐1‐Ser* may be involved in the regulation of RNA alternative splicing activities mediated by 25(OH)D.

In conclusion, *tRF‐1‐Ser* plays a crucial role in the progression of breast cancer by participating in the regulation of diverse biological activities.

### 3.4 *tRF‐1‐Ser* can tightly bind to MBNL1 through the nucleotide sequence UGCU

**FIGURE 4:**
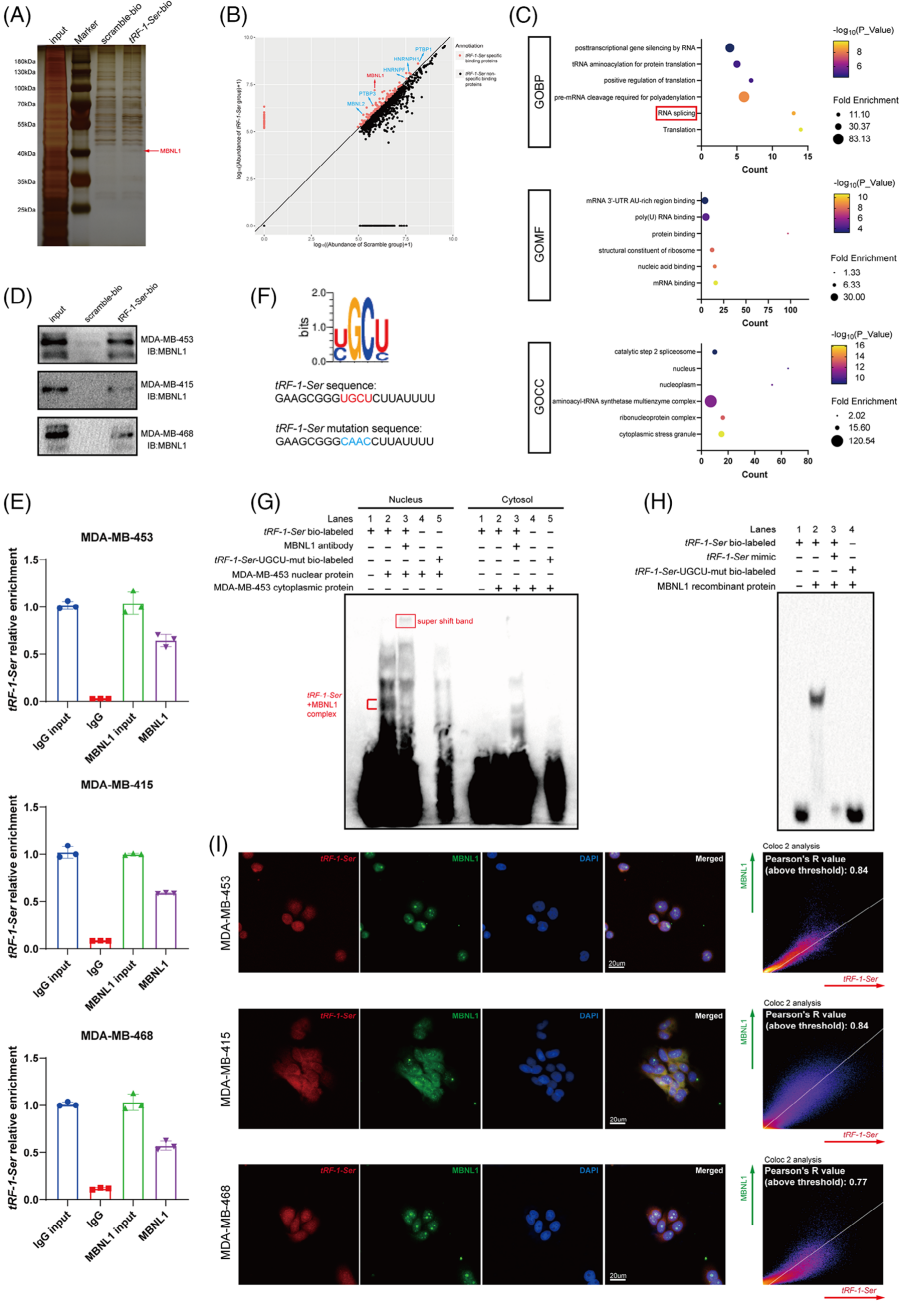
tRF‐1‐Ser can tightly bind to MBNL1 through the nucleotide sequence ‘UGCU’. (A) The protein sliver staining in MDA‐MB‐453 pulled down by tRF‐1‐Ser or scramble RNA was observed to have a slight variation. (B) A scatter plot showed the abundance of proteins pulled down by tRF‐1‐Ser or scramble, which were identified through high‐performance liquid chromatography–mass spectrometry/mass spectrometry (HPLC–MS/MS) and transformed into log10. The protein abundance ratio of tRF_G/S_G > 1.5 was marked in red and referred to as specific binding proteins (a total of 110 proteins). tRF_G, tRF‐1‐Ser pull‐down proteins group. S_G, scramble RNA pull‐down proteins group. (C) The specific binding proteins were analysed with Gene Ontology on DAVID, and some of the results were displayed in bubble plots. (D) The interaction of tRF‐1‐Ser in MDA‐MB‐453, MDA‐MB‐415 and MDA‐MB‐468 was confirmed through an independent RNA pull‐down assay followed by a Western blotting (WB) assay. (E) The binding capacity of MBNL1 to tRF‐1‐Ser was confirmed through an RNA immunoprecipitation (RIP) assay with MBNL1 antibody followed by qPCR. U6 was applied as a control of qPCR. (F) The RBPDB was used to predict the binding motif of tRF‐1‐Ser, and the original and mutant sequences were displayed. (G) Electrophoretic mobility shift assay (EMSA) assay was performed to evaluate the binding ability of tRF‐1‐Ser and tRF‐1‐Ser‐UGCU‐mut with nuclear and cytoplasmic proteins. MBNL1 antibody was applied to confirm that tRF‐1‐Ser can bind with MBNL1. (H) EMSA assay was also carried out to assess the binding ability of tRF‐1‐Ser and tRF‐1‐Ser‐UGCU‐mut with MBNL1 recombinant protein. tRF‐1‐ Ser mimic was applied to explore the competitive binding relationship between biotin‐labelled tRF‐1‐Ser and tRF‐1‐Ser mimic. (I) Fluorescence in situ hybridisation (FISH) and immunofluorescence (IF) assays were carried out to confirm the subcellular location and the colocalisation of tRF‐1‐Ser and MBNL1. Data are shown as mean ± standard deviation (SD). ^*^ *p* < .05, ^**^ *p* < .01, ^***^ *p* < .001.

The most extensively reported mechanism of action of tsRNAs involves directly binding to RBPs to modulate their functions and thereby participate in the biological regulation of cancer. To further explore this mechanism, we labelled *tRF‐1‐Ser* and scrambled RNA with biotin and incubated them with MDA‐MB‐453 cell lysates for RNA pull‐down. The precipitated products were electrophoretically separated using SDS–PAGE, and proteins were visualised using silver staining (Figure 4A). The results revealed significant differences in the binding proteins between scramble and *tRF‐1‐Ser*.

HPLC–MS/MS was employed to identify pull‐down products, designating the products from *tRF‐1‐Ser* pull‐down as tRF_G and those from scramble RNA pull‐down as S_G. Protein abundances of S_G and tRF_G were logarithmically transformed and represented in a scatter plot. Proteins with a tRF_G/S_G abundance ratio >1.5 (a total of 110 proteins) were highlighted in red (Figure 4B) and categorised as *tRF‐1‐Ser*‐specific binding proteins. Subsequently, enrichment analysis on the *tRF‐1‐Ser*‐specific binding proteins was performed using DAVID,^22^, ^23^ an online gene annotation and analysis database (Figure 4C) (Table S2). Among the results of the GO analysis, the most prominent categories include translation, tRNA aminoacylation for protein translation and RNA splicing. Of particular interest is RNA splicing, as this term emerged consistently in the results of GSEA from the transcriptomic data obtained after 25(OH)D cultivation and *tRF‐1‐Ser* knockdown (Figures 2B and 3F). We annotated proteins related to RNA splicing in the mass spectrometry results (Figure 4B), revealing that the tRF_G /S_G value of MBNL1 was significantly higher than that of other proteins, suggesting that *tRF‐1‐Ser* may predominantly influence the function of MBNL1.

To confirm our hypothesis, we initially performed RNA pull‐down experiments in the remaining two cell lines, MDA‐MB‐415 and MDA‐MB‐468. Alongside Western blot analysis, we observed that the interaction between *tRF‐1‐Ser* and MBNL1 was not an isolated phenomenon (Figure 4D). Subsequently, RIP‐qPCR revealing that the *tRF‐1‐Ser* precipitated using the MBNL1 antibody group was significantly higher than that in the immunoglobulin G antibody group across all three cell lines (Figure 4E).

The RBPDB database^24^ was used to predict the RBPs that *tRF‐1‐Ser* may bind to, among which MBNL1 was identified (Figure S3D). According to these predictions, *tRF‐1‐Ser* may interact with MBNL1 through the UGCU base motif (Figure 4F). Reports also suggest that MBNL1 exhibits a high affinity for the YGCY base motif.^25^

We conducted electrophoretic mobility shift assay (EMSA) with biotin‐labelled *tRF‐1‐Ser* and *tRF‐1‐Ser*‐mutation, where we mutated the UGCU motif. Nuclear and cytoplasmic proteins were isolated from MDA‐MB‐453 cells, revealing that *tRF‐1‐Ser* had a higher binding affinity with nuclear‐extracted proteins (Figure 4G), which is consistent with our GO analysis (Figure 4C). The binding ability significantly decreased after mutation of the UGCU motif, and the addition of MBNL1 antibody during incubation led to a supershift band. The binding band below lane 3 exhibited obvious attenuation (Figure 4G), suggesting an interaction between *tRF‐1‐Ser* and MBNL1.

To explore the binding relationship between *tRF‐1‐Ser* and MBNL1, we incubated MBNL1 recombinant protein with biotin‐labelled *tRF‐1‐Ser* and *tRF‐1‐Ser*‐mutation. We established a competitive binding group with excess unlabelled *tRF‐1‐Ser*. The EMSA results showed tight binding between MBNL1 and *tRF‐1‐Ser*, which disappeared upon the addition of excess unlabelled *tRF‐1‐Ser*. Furthermore, MBNL1 did not bind to the mutated *tRF‐1‐Ser* without the UGCU motif (Figure 4H).

We also conducted FISH and IF experiments targeting *tRF‐1‐Ser* and MBNL1, capturing images using confocal fluorescence microscopy. Colocalisation analysis with ImageJ revealed a high degree of spatial overlap between the two, with MBNL1 primarily distributed in the cell nucleus, whereas *tRF‐1‐Ser* exhibited a comparable distribution in both the nucleus and cytoplasm (Figure 4I).

### 3.5 *tRF‐1‐Ser* regulates RNA splicing by inhibiting the nuclear transportation of MBNL1

**FIGURE 5:**
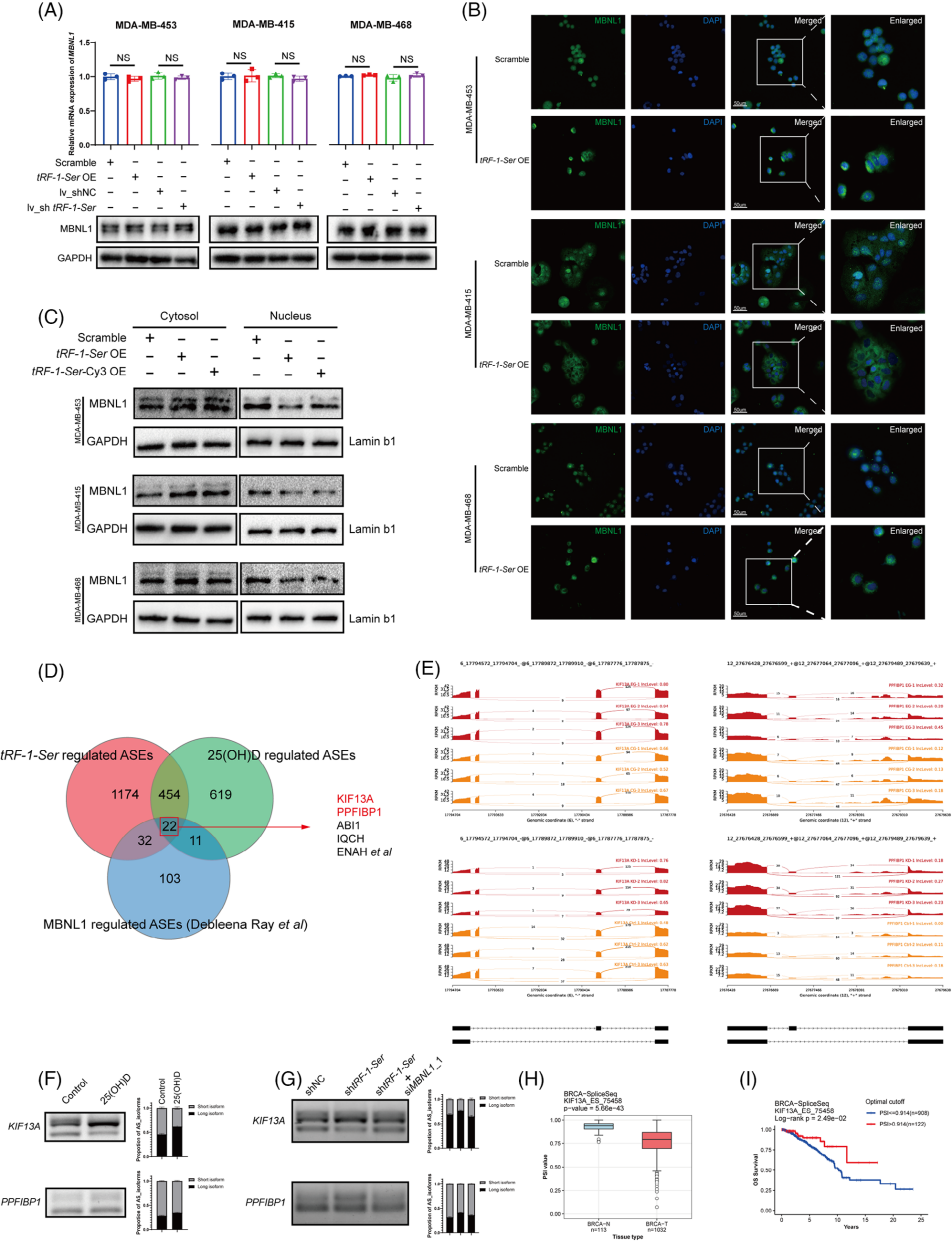
tRF‐1‐Ser regulates RNA splicing by inhibiting the nuclear transportation of MBNL1. (A) The overexpression or knockdown of tRF‐1‐Ser did not influence the mRNA or protein levels of MBNL1. U6 was applied as a control of qPCR. (B) Immunofluorescence (IF) assay was conducted to assess the change in the subcellular location of MBNL1. (C) The cytoplasmic and nuclear proteins of BRCA cells overexpressed tRF‐1‐Ser were separated and the protein level of MBNL1 was detected by immunoblot analysis. Lamin b1 was used as a nuclear control, and GAPDH was applied as a cytoplasmic control. (D) Venn diagram depicted the interaction of alternative splicing events (ASEs) regulated by 25‐hydroxyvitamin D (25(OH)D), tRF‐1‐Ser and MBNL1. (E) The ASEs regulated by 25(OH)D, tRF‐1‐Ser and MBNL1 were shown with sashimi plot. (F) The ASEs of exon 26 in KIF13A and exon 19 in PPFIBP1 were detected by semi‐quantitative PCR. Gel electrophoresis images are on the left and quantitative bar graphs analysed by ImageJ are on the right. The total RNA was obtained from MDA‐MB‐453 cultured with 25(OH)D (F) or transfected with shtRF‐1‐Ser or siMBNL1 (G). (H) The percentage spliced in (PSI) of KIF13A_ES_75458 in normal or BRCA tissues was analysed on the OncoSplicing database. An unpaired *t*‐test was applied. (I) The survival curve was conducted to assess the influence of KIF13A_ES_75458 by the OncoSplicing database. KIF13A_ES_75458, the name of the event of exon 26 skipping in KIF13A assigned by the OncoSplicing database. ^*^ *p* < .05, ^**^ *p* < .01, ^***^ *p* < .001, NS means ‘not statistically significant’.

Despite the significant binding affinity observed between *tRF‐1‐Ser* and MBNL1, our initial exploration into the impact of *tRF‐1‐Ser* expression changes on MBNL1 expression yielded unexpected results. Knockdown and overexpression of *tRF‐1‐Ser* did not influence the mRNA and protein expression levels of MBNL1 (Figure 5A).

We fluorescently labelled *tRF‐1‐Ser* with Cy3 and transfected it into three breast cancer cell lines. On the third day post‐transfection, we observed that exogenous *tRF‐1‐Ser* was predominantly localised in the cytoplasm of the cells (Figure S4B). The result we obtained was distinct from the phenomenon we observed in our FISH–IF dual staining, suggesting the limited nuclear entry of *tRF‐1‐Ser*; this agrees with literature reports indicating that tsRNAs can affect the nucleocytoplasmic transport of their binding proteins.^26^ Therefore, *tRF‐1‐Ser*, which resides in the cytoplasm, was hypothesised to affect the nuclear transportation of its binding proteins. Subsequently, after *tRF‐1‐Ser* overexpression, an IF assay revealed altered fluorescent localisation of MBNL1, predominantly concentrated in the cytoplasm (Figure 5B). After separating nuclear and cytoplasmic proteins, Western blot experiments confirmed that MBNL1 expression was increased in the cytoplasm and decreased in the nucleus (Figure 5C).

It has been reported that the inclusion of exon 5 in MBNL1 plays a crucial role in its nuclear localisation, and MBNL1 possesses a subtle autoregulatory system (Figure S4I) whereby MBNL1 protein can adjust its splicing function by promoting exclusion of exon 5 in MBNL1 pre‐mRNA.^27^ We proposed two hypothesis to explain the abnormal cytoplasmic location of MBNL1. One is that *tRF‐1‐Ser* may interfere with the normal autoregulatory system of MBNL1, leading to increased expression of MBNL1△exon 5, thereby causing MBNL1 to primarily localise in the cytoplasm (Figure S4J). The other is that *tRF‐1‐Ser* disrupt the nuclear entry pathway of MBNL1 protein, resulting in abnormal cytoplasmic accumulation of MBNL1 and loss of its splicing function (Figure S4K). To distinguish between these two hypotheses, we examined alternative splicing events of exon 5 in *MBNL1*. Surprisingly, the results showed a significant decrease in MBNL1△exon 5 expression after *tRF‐1‐Ser* overexpression (Figure S4H). This contradicts the premise of hypothesis one, thus indicating that hypothesis two is more possible.

Previous studies have identified MBNL1 as an RBP with a C3H zinc finger structure, which plays a crucial role in various RNA processing steps, particularly in regulating alternative RNA splicing by acting as a splicing factor.^28^, ^29^ Pre‐mRNA splicing always occurs in the nucleus of eukaryotes, whereas spliceosome assembly and splicing factors regulate alternative RNA splicing within the nucleus. Therefore, we hypothesise that *tRF‐1‐Ser* regulates alternative splicing by impeding the entry of MBNL1 into the nucleus. To validate this hypothesis, we analysed the genes involved in alternative splicing events possibly regulated by 25(OH)D and *tRF‐1‐Ser* using the rmats software^30^, ^31^, ^32^ on two consecutive RNA‐seq datasets (Table S3). These events were compared with those reported by Ray et al. using a Venn diagram, indicating genes regulated by MBNL1^29^ (Figure 5D). The results revealed 22 genes under common regulation by all three factors. Upon further comparison of the splice sites and regulatory directions, KIF13A and PPFIBP1 aligned completely with our speculation (Figure 5E). Subsequently, we established an MBNL1 knockdown model in MDA‐MB‐453 (Figure S4A), and semi‐quantitative PCR confirmed the expression changes in alternative splicing isoforms of KIF13A, PPFIBP1 and MAP2K7. MAP2K7 was chosen because of its significant regulation by MBNL1 in the study by Ray et al. After MBNL1 knockdown, we found varying degrees of exon skipping for KIF13A exon 26, PPFIBP1 exon 19 and MAP2K7 exon 2 (Figure S4C). Simultaneously, we validated that under 25(OH)D cultivation, exon skipping events in KIF13A exon 26, PPFIBP1 exon 19 and MAP2K7 exon 2 all decreased (Figures 5F and S4D). After knocking down *tRF‐1‐Ser* expression, exon skipping events for these three genes similarly decreased. Interestingly, when both *tRF‐1‐Ser* and MBNL1 were knocked down simultaneously, exon skipping events increased (Figures 5G and S4E). These observations further validate our sequencing results and hypotheses. We analysed the effects of different splicing events using the OncoSplicing public database,^33^, ^34^ revealing that the percentage spliced in (PSI) values for KIF13A exon 26 and MAP2K7 exon 2 were higher in normal breast tissue than in breast cancer tissue (Figures 5H and S4F). Additionally, higher PSI values for KIF13A exon 26 and MAP2K7 exon 2 were associated with better overall survival rates in patients (Figures 5I and S4G). Therefore, *tRF‐1‐Ser* regulates alternative splicing by inhibiting the nuclear entry of MBNL1, leading to a malignant shift in mRNA alternative splicing isoforms, and 25(OH)D reduces this alternative splicing trend by downregulating *tRF‐1‐Ser*.

### 3.6 *tRF‐1‐Ser* enhances tumour stemness by inhibiting negative regulation of the JNK pathway by MBNL1

The results of our experimental study lead us to believe that *tRF‐1‐Ser* can hinder the functional role of MBNL1. MBNL1 plays a crucial role in regulating RNA splicing patterns during embryonic stem cell differentiation, which has been extensively studied and is well established.^28^, ^35^ In malignant tumours, including breast cancer, MBNL1 expression is often reduced. Additionally, it has been reported that MBNL1 suppresses the JNK pathway, which in turn reduces the stemness of breast cancer cells.^29^

**FIGURE 6:**
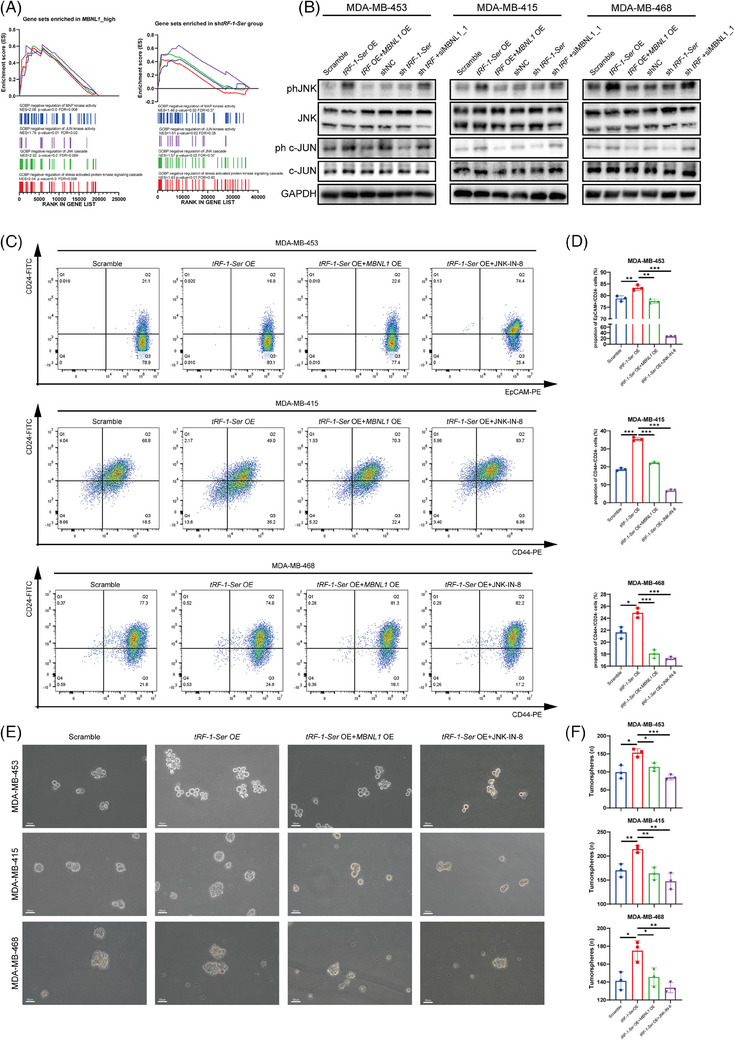
tRF‐1‐Ser enhances tumour stemness by inhibiting the negative regulation of the JNK pathway by MBNL1. (A) Gene set enrichment analysis (GSEA) further showed the function of MBNL1 or tRF‐1‐Ser in regulating the JNK cascade and JUN kinase activity. A total of 1231 BRCA cases from The Cancer Genome Atlas (TCGA) were used to analyse the enrichment pathway of MBNL1. (B) Western blotting (WB) assay was carried out to confirm the regulatory relationship between tRF‐1‐Ser and MBNL1 about the activation of JNK and c‐JUN. GAPDH was applied as the control of WB. (C) Flow cytometry was used to detect the change in the percentage of breast cancer stem cells (BCSCs) in MDA‐MB‐453, MDA‐MB‐415 and MDA‐MB‐468. Cells were overexpressed tRF‐1‐Ser alone or overexpressed both tRF‐1‐Ser and MBNL1 or overexpressed tRF‐1‐Ser and cultured in 1 μM JNK‐IN‐8. (D) Bar graphs show the percentage of BCSCs from three independent experiments. (E) Tumour‐sphere assay was conducted to assess the stemness of BRCA cells and representative images at 20× magnification were photoed. (F) Tumour spheres with a diameter larger than 50 μm were counted and the bar graphs show the number from three independent experiments. Data are shown as mean ± standard deviation (SD). An unpaired *t*‐test was applied to calculate the significance of two groups. ^*^ *p* < .05, ^**^ *p* < .01, ^***^ *p* < .001.

To understand how *tRF‐1‐Ser* and MBNL1 interact and regulate biological functions, we analysed transcriptome data from TCGA containing 1231 breast cancer cases, categorised based on the average TPM values of MBNL1 into high‐ and low‐expression groups (MBNL1_high and MBNL1_low, respectively). We then compared the biological processes between the two groups using the GSEA algorithm with the GOBP dataset. Our findings suggest that MBNL1 negatively regulates the JNK cascade, c‐JUN kinase activity and MAPK kinase activity (Figure 6A). Interestingly, similar pathways were also enriched in the *tRF‐1‐Ser* knockdown group (Figure 6A). These results provide new insights into the potential role of MBNL1 and *tRF‐1‐Ser* in breast cancer.

We hypothesised that *tRF‐1‐Ser* can activate the JNK cascade by inhibiting the negative regulation of JNK activity by MBNL1. To test this hypothesis, we initially knocked down MBNL1 in MDA‐MB‐453 and verified that the JNK pathway was activated (Figure S5B). We then investigated the effects of both *tRF‐1‐Ser* and MBNL1 on the JNK cascade. Our findings indicate that *tRF‐1‐Ser* overexpression enhances JNK activity, which is subsequently diminished by MBNL1 overexpression (Figure 6B), and vice versa. Considering the roles of MBNL1 and JNK‐in BCSCs, we conducted flow cytometry and sphere formation assays to study the effects of *tRF‐1‐Ser* on breast cancer stemness. CD44 and CD24 are commonly used markers for BCSCs, with CD44+/CD24‒ cells representing a stronger tumour stemness.^36^ Considering that MDA‐MB‐453 lacks CD44 expression and research indicates that EpCAM+/CD24‒ MDA‐MB‐453 cells possess cancer stem cell characteristics, we used EpCAM and CD24 as markers to identify the proportion of BCSCs in MDA‐MB‐453.^37^ Our results demonstrate that upon *tRF‐1‐Ser* overexpression, the proportion of BCSCs exhibits varying degrees of upregulation, which can be reversed with the MBNL1 overexpression. Moreover, the proportion of BCSCs was significantly reduced with *tRF‐1‐Ser* overexpression and treatment with JNK‐IN‐8 (1 μM), a potent JNK inhibitor (Figure 6C,D). Similar findings were observed in the sphere formation assays. The overexpression of *tRF‐1‐Ser* increased the sphere‐forming ability of breast cancer cells, as evidenced by a significant increase in the number of spheres, which was countered by the overexpression of MBNL1 and the use of JNK‐IN‐8 (Figure 6E,F). Therefore, *tRF‐1‐Ser* can enhance the stemness of breast cancer cells by inhibiting the negative regulation of JNK activity by MBNL1.

### 3.7 Inhibition of *tRF‐1‐Ser* plays a crucial role in the anticancer effects of 25(OH)D

**FIGURE 7:**
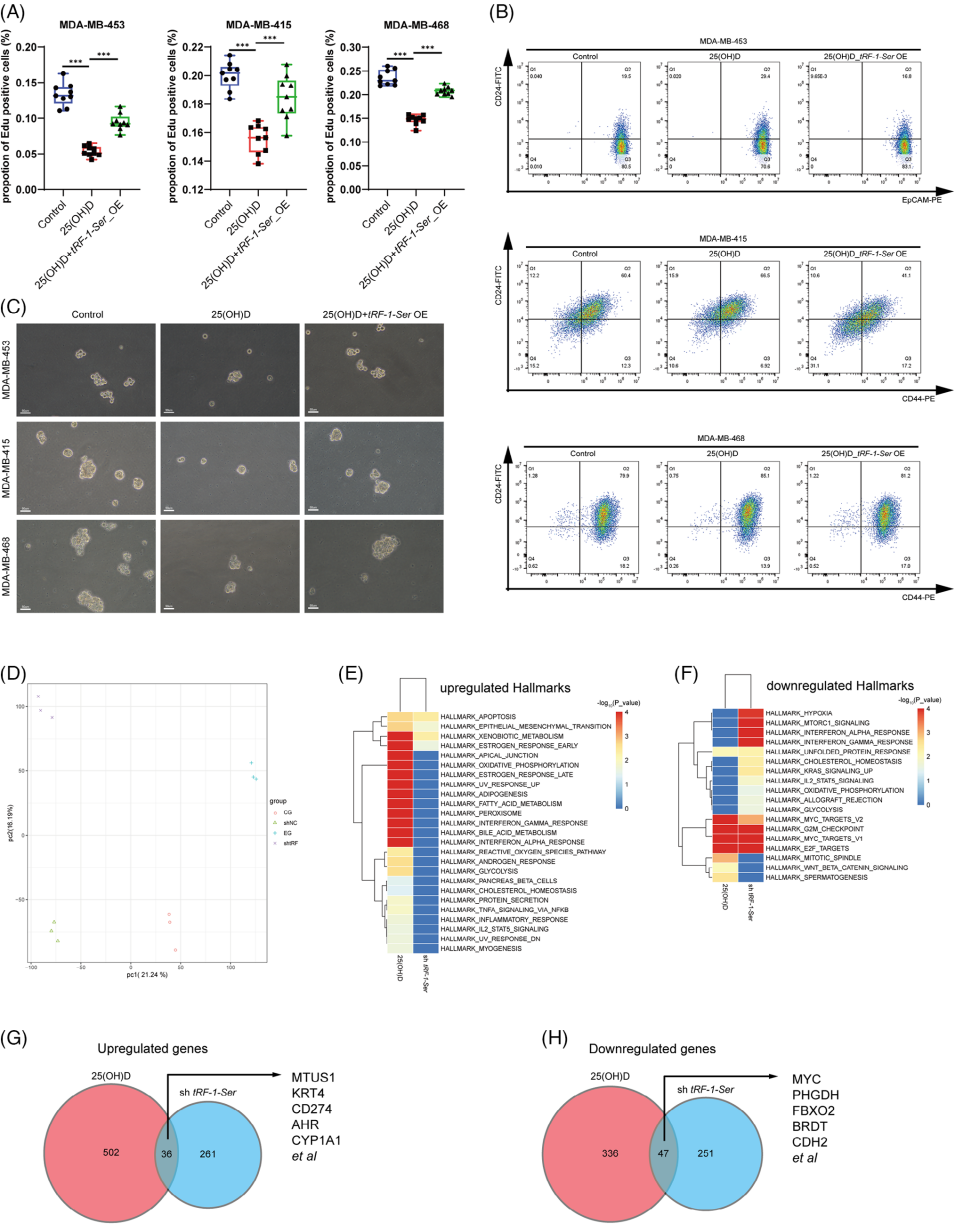
Inhibition of tRF‐1‐Ser plays a crucial role in the anticancer effects of 25‐hydroxyvitamin D (25(OH)D). (A) EDU assay was performed to detect the influence of the rescued experiment. (B) Flow cytometry was used to detect the change in the percentage of breast cancer stem cells (BCSCs) in the rescued experiment. (C) Tumour‐sphere assay was conducted to assess the stemness of BRCA cells in the rescued experiment and representative images at 20× magnification were photoed. (D) PCA was performed to ensure the quality of our transcriptome data. CG, control group of 25(OH)D; EG, experiment group refers to MDA‐MB‐453 cultured in 100 nM 25(OH)D; shNC, MDA‐MB‐453 transfected with shNC; shtRF, MDA‐MB‐453 transfected with shtRF‐1‐Ser. (E) The upregulated and (F) downregulated Hallmarks of 25(OH)D and shtRF‐1‐Ser analysed by gene set enrichment analysis (GSEA) were shown with a heatmap. Venn diagrams depicted (G) the common upregulated genes or (H) the downregulated genes. An unpaired *t*‐test was applied to calculate the significance of two groups. ^*^ *p* < .05, ^**^ *p* < .01, ^***^ *p* < .001.

To further elucidate the role of *tRF‐1‐Ser* in the antitumour effects of 25(OH)D, rescue experiments were conducted in the context of its anticancer effects. After treatment of breast cancer cells with 25(OH)D, *tRF‐1‐Ser* was overexpressed, and changes in cell proliferation and stemness were assessed. Reintroduction of *tRF‐1‐Ser* significantly reduced the antiproliferative capacity of 25(OH)D (Figure 7A). Flow cytometry revealed that 25(OH)D significantly downregulated the proportion of EpCAM+/CD24‒ cells in MDA‐MB‐453 cells. In MDA‐MB‐415 and MDA‐MB‐468 cells, 25(OH)D reduced the proportion of CD44+/CD24‒ cells (Figure 7B). Sphere formation assays revealed a similar trend, where 25(OH)D significantly diminished the sphere‐forming ability of breast cancer cells (Figure 7C). The overexpression of *tRF‐1‐Ser* reversed all these phenomena. These phenomena further underscore the crucial role of *tRF‐1‐Ser* downregulation in the anticancer effects of 25(OH)D.

We also conducted transcriptome data analysis for 25(OH)D and *tRF‐1‐Ser* knockdown. Principal components analysis (PCA) analysis of the four sets of transcriptome data showed minimal intragroup differences and some intergroup distinctions, affirming the scientific validity of our transcriptome data (Figure 7D). GSEA using the HALLMARK dataset revealed a partial overlap in enriched pathways between the 25(OH)D and *tRF‐1‐Ser* knockdown groups (Figure 7E,F). The downregulation of pathways such as MYC_TARGETS, G2M_CHECKPOINT and E2F_TARGETS under both conditions was particularly interesting. Furthermore, in terms of differentially expressed genes, the two sets of transcriptome data exhibited some commonly regulated genes (Figure 7G,H).

### 3.8 *tRF‐1‐Ser* promotes tumour growth in vivo

**FIGURE 8:**
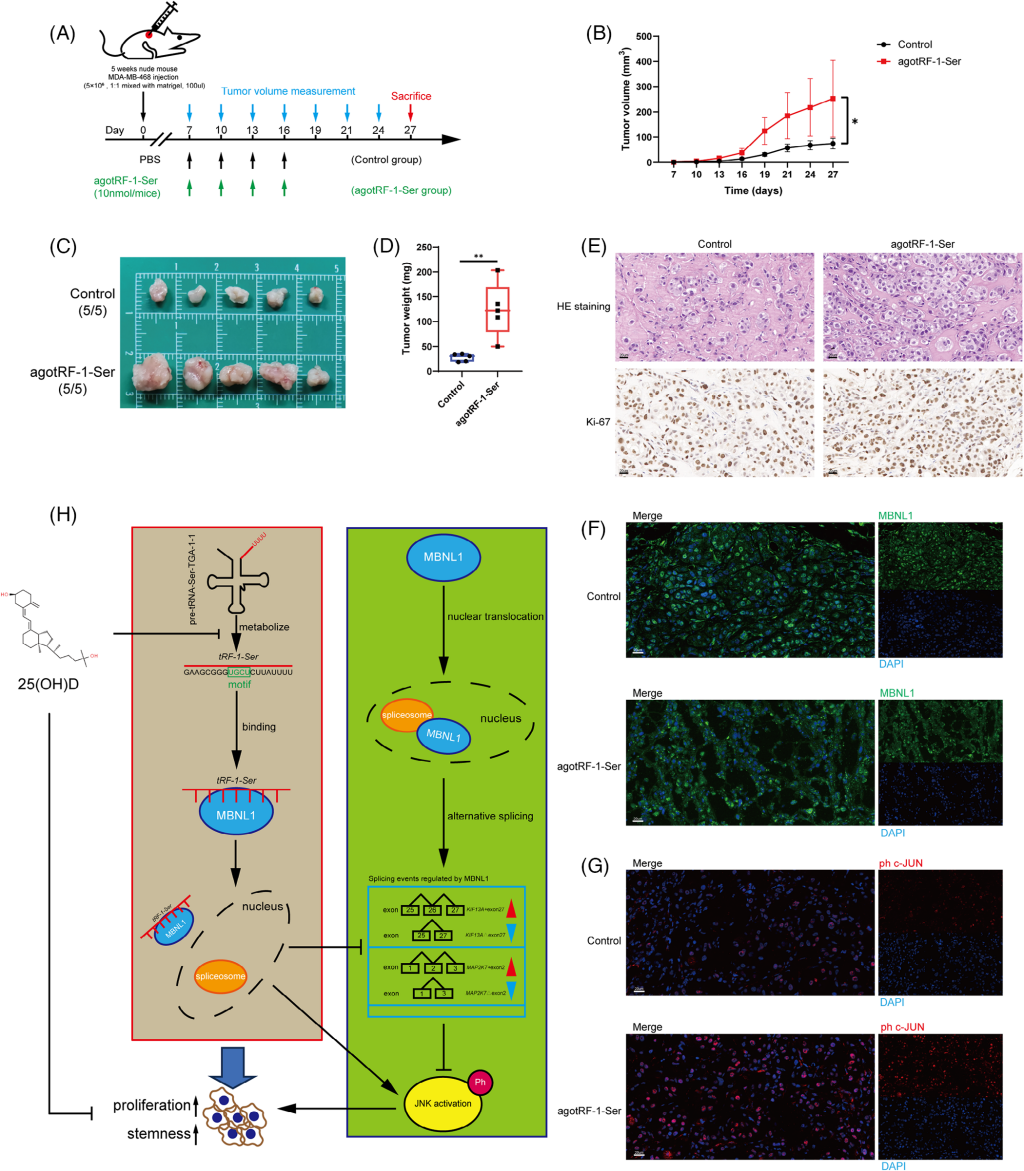
tRF‐1‐Ser promotes tumour growth in vivo. (A) The workflow shows the construction of tRF‐1‐Ser overexpression subcutaneous xenograft models. (B) The growth curves of subcutaneous tumours were shown. Two‐way analysis of variance (ANOVA) was performed to calculate the significance of two groups. (C) The tumours dissected from nude mice were photoed and (D) weighted. (E) Haematoxylin–eosin (HE) staining (up) of tumours confirmed the successful construction of xenograft models. Immunohistochemistry (IHC) (down) of tumours indicated a higher proportion of ki‐67‐positive cells in agotRF‐1‐Ser group. (F) Immunofluorescence (IF) assay indicated the change in the subcellular location of MBNL1 in the agotRF‐1‐Ser group. (G) The IF assay showed a higher expression of ph c‐JUN in the agotRF‐1‐Ser group. Data are shown as mean ± standard deviation (SD). An unpaired *t*‐test was applied to calculate the significance of two groups. ^*^ *p* < .05, ^**^ *p* < .01, ^***^ *p* < .001.

A subcutaneous xenograft model was established in BALB/c nude mice using MDA‐MB‐468 cells. Two groups were established: the ago*tRF‐1‐Ser* and CGs. The former was injected with ago*tRF‐1‐Ser* to simulate *tRF‐1‐Ser* overexpression conditions, whereas the CG was injected with PBS (Figure 8A). Tumour dimensions were measured every 3 days using callipers. A significantly faster subcutaneous tumour growth rate was seen in the ago*tRF‐1‐Ser* group versus the CG (Figure 8B). Subcutaneous tumours were excised, photographed and weighed at the end of week 4 (Figure 8C,D). The ago*tRF‐1‐Ser* group had notably heavier tumours than the CG, suggesting that *tRF‐1‐Ser* promotes the development of breast tumours in vivo. Afterward, HE staining and IHC were performed on the subcutaneous tumours. The results showed a significant increase in the proportion of ki‐67‐stained cells in the ago*tRF‐1‐Ser* group (Figure 8E). We also performed IF experiments to assess the subcellular localisation and expression levels of MBNL1 and ph c‐JUN. The subcellular localisation of MBNL1 differed between the two groups, being predominantly localised nucleus and cytoplasm, respectively, in the CG and ago*tRF‐1‐Ser* group, respectively (Figure 8F). Moreover, there was a significant increase in ph c‐JUN expression in the ago*tRF‐1‐Ser* group (Figure 8G).

Stable MDA‐MB‐468 cell lines were transfected with sh*tRF‐1‐Ser* and shNC by lentiviral vectors in vivo (Figure S7A). The tumourigenic capacity of the sh*tRF‐1‐Ser* group was significantly reduced, accompanied by a decelerated tumour growth rate (Figure S7B,C).

## 4 DISCUSSION

An increasing body of evidence has analysed the association between nutritional status and the risk and prognosis of cancer.^38^ Many studies have investigated the relevance of vitamin D and its metabolites to the occurrence and progression of breast cancer. However, despite compelling evidence demonstrating the significant anticancer effects of vitamin D and its metabolites,^2^, ^3^, ^4^ the conclusions of clinical research conclusions remain unclear.^5^, ^6^, ^7^, ^8^, ^9^ While assessing serum 25(OH)D levels in BRCA patients, we observed that the majority exhibited subnormal levels of serum 25(OH)D (Figure S1A), suggesting importance of exploring the effect of insufficient serum 25(OH)D levels on BRCA patients. The anticancer effects of 25(OH)D are exhibited by its conversion to 1,25(OH)2D through both paracrine and autocrine mechanisms. However, this effect diminishes upon CYP27B1 knockout^12^ and is also affected by VDR expression.^39^ These observations guided our selection of breast cancer cell lines sensitive to 25(OH)D, suggesting that the sensitivity to 25(OH)D may vary significantly due to the heterogeneity of breast cancer, potentially contributing to inconsistent conclusions in clinical research. Consequently, our research aims to discover downstream biomarkers regulated by 25(OH)D in breast cancer cells to assist in further understanding the mechanism of 25(OH)D in breast cancer.

Our transcriptome data revealed that 25(OH)D modulates the metabolic processes of tRNA, a phenomenon also documented by Rabbani and coworkers.^19^ Given the increasing significance of tsRNAs in clinical diagnostics,^40^, ^41^ we conducted sequencing and screening of tsRNAs regulated by 25(OH)D. Our results revealed that *tRF‐1‐Ser* exhibited the most pronounced negative regulation by 25(OH)D. The sequence of *tRF‐1‐Ser* is highly similar to that of tRF‐1001, which is reportedly highly expressed in prostate cancer and promotes its proliferation.^17^ Similarly, our in vivo and in vitro studies demonstrated the promotive effect of *tRF‐1‐Ser* on cancer cell proliferation in breast cancer. Subsequently, we found a group of proteins bound to *tRF‐1‐Ser* through RNA pull‐down experiments combined with HPLC–MS/MS. Enrichment analysis using DAVID revealed that these proteins were involved in RNA splicing regulation. Notably, the most abundantly bound protein was MBNL1, which regulates RNA splicing and induces stem cell differentiation.^28^ Previous SELEX experiments demonstrated that MBNL1 recognises and binds to YGCY motifs,^25^ which are in the nucleotide sequence of *tRF‐1‐Ser*. This interaction was further confirmed through EMSA experiments, which strongly suggested that *tRF‐1‐Ser* functions by binding to MBNL1.

MBNL1 is a splicing factor that regulates RNA splicing and enhances early spliceosome assembly in the nucleus^42^ and the dysregulation of MBNL1 function and expression is linked to cancer development. In breast cancer, MBNL1 negatively regulates the JNK pathway, thus inhibiting the stemness of breast cancer by modulating RNA splicing.^29^ In malignant gliomas, hypoxic conditions impede the nuclear transport of MBNL1 by strengthening the exclusion of exon 5 of MBNL1, thereby promoting glioma cell stemness.^43^ In our study, *tRF‐1‐Ser* overexpression inhibited the nuclear transport of MBNL1. Our hypothesis suggests that *tRF‐1‐Ser* has the potential to disrupt the pathway for nuclear entry of the MBNL1 protein, resulting in an atypical cytoplasmic accumulation of MBNL1. This accumulation, in turn, leads to the loss of splicing function. However, we did not dig into the molecular mechanism by which *tRF‐1‐Ser* affects MBNL1 nuclear transport because there is currently limited research on protein interactions related to MBNL1 nuclear transport in cancer. We need to design more rigorous experiments to explore the mechanism of MBNL1 nuclear transport. Transcriptome data for 25(OH)D and sh*tRF‐1‐Ser* showed changes in RNA subtypes regulated by MBNL1. GSEA of public transcriptome data also identified associations between elevated MBNL1 expression and downregulation of the JNK cascade and c‐JUN kinase activity. This regulatory effect was also evident in the GSEA of the sh*tRF‐1‐Ser* group. These findings were validated via Western blot experiments. Furthermore, *tRF‐1‐Ser* enhanced breast cancer stemness, which can be reversed by the MBNL1 overexpression or JNK‐IN‐8 application.

After confirming the mechanisms and function of *tRF‐1‐Ser*, we investigated the impact of its downregulation on the anticancer effects of 25(OH)D, revealing that *tRF‐1‐Ser* boosts the stemness breast cancer cells and their ability to form spheres. We also investigated the association between 25(OH)D and BCSCs, revealing that 25(OH)D significantly reduced the proportion of BCSCs and their ability to form spheres, consistent with previous research.^3^, ^44^, ^45^

Our study focused on 25(OH)D, whereas earlier studies mainly used 1,25(OH)2D. Our rescue experiments revealed that *tRF‐1‐Ser* overexpression could mitigate the inhibitory effects of 25(OH)D on breast cancer proliferation and stemness. Transcriptome analyses of the 25(OH)D and sh*tRF‐1‐Ser* groups also revealed shared regulatory pathways, including MYC_Targets, G2M_Checkpoint and E2F_Targets. Furthermore, both entities displayed a shared regulation of genes, including MYC, PHGDH, FBXO2 and BRDT, which promote cancer progression according to previous studies. It is noteworthy that numerous studies have extensively reported the inhibitive effect of 1,25(OH)_2_D on MYC.^46^, ^47^ Building upon these findings, we conducted an analysis of MYC expression following treatment with 25(OH)D or sh*tRF‐1‐Ser* (Figure S6F), and the results align with our transcriptome analyses. These findings provide strong evidence supporting our hypotheses.

Our team conducted a comprehensive study to explore the potential diagnostic and therapeutic benefits of *tRF‐1‐Ser*. We collected postoperative samples from 62 breast cancer patients with their consent and ethics approval. Each patient had a clear diagnosis before surgery, and standard pathological examinations were performed afterward. Absolute quantitative PCR (Figure S2F,G) allowed us to measure *tRF‐1‐Ser* levels in breast cancer tissues and their corresponding normal tissues. Our findings revealed that *tRF‐1‐Ser* expression was higher in tumour tissues than in normal tissues. In addition, we correlated the expression levels of *tRF‐1‐Ser* in tumour tissues with clinical data from the patients, revealing a correlation between *tRF‐1‐Ser* levels and tumour size (Table 1). While we did not find a correlation between *tRF‐1‐Ser* and serum 25(OH)D levels, we did identify a weak negative correlation when we analysed their linear relationship (Figure S2H). Nevertheless, further studies with a larger sample size is needed to validate our findings.

We still have some unsolved puzzles in our study. Recent research has suggested that the methylation of tRF‐3s may influence gene expression and the unfolded protein response.^48^ This implies that the base modification of tRFs has its own unique biological function that is yet to be explored. In order to simulate the overexpression of *tRF‐1‐Ser*, we used *tRF‐1‐Ser* mimic, which has been acknowledged by numerous research articles.^21^, ^26^, ^49^ However, the drawback of this method is that tRFs mimics may lack potential natural base modification, which means that we may miss some other functions of *tRF‐1‐Ser*. The field of base modification of tRFs is a fascinating and complex area that is yet to be fully explored. Unfortunately, there is still a long way to go in understanding the mechanism of tRFs base modification.

Overall, our study offers a novel understanding of how 25(OH)D exerts its anticancer effects through tsRNAs, revealing the regulatory relationship between 25(OH)D and *tRF‐1‐Ser*, as well as the mechanistic actions of *tRF‐1‐Ser* with MBNL1. Our findings provide insights into downstream products regulated by 25(OH)D, which can guide the clinical treatment of breast cancer patients with 25(OH)D deficiency. Ultimately, *tRF‐1‐Ser* can be a therapeutic target in breast cancer.

## AUTHOR CONTRIBUTIONS

Xinyu Wan, Wenjie Shi, Lingjun Ma, and Lexin Wang are responsible for the design of thestudy and the conduct of experiments. Ran Zheng and Jingzhi He are accountablefor analyzing clinical samples and information. Collection of clinical samples:Ye wang and Xuan Li. The draft and revision of the manuscript: Xiaoming Zha,Jue Wang, and Lu Xu.

## CONFLICT OF INTEREST STATEMENT

The authors declare they have no conflicts of interest.

## ETHICS STATEMENT

The collection of clinical samples was approved by the Ethics Committee of Jiangsu Province Hospital (ethics review number: 2023‐SR‐977). All animal experiments were approved by the Institutional Animal Care and Use Committee of Nanjing Medical University (ethics review number: IACUC‐2305006).

## Supporting information

Supporting Information

Supporting Information

Supporting Information

Supporting Information

Supporting Information

Supporting Information

Supporting Information

Supporting Information

Supporting Information

Supporting Information

## Data Availability

The datasets used during the current study are available from the corresponding author on reasonable request.
