# Supplementary figures and images for: A 3′‐pre‐tRNA‐derived small RNA tRF‐1‐Ser regulated by 25(OH)D promotes proliferation and stemness by inhibiting the function of MBNL1 in breast cancer
Source: Clin Transl Med. 2024 May 9;14(5):e1681. doi: 10.1002/ctm2.1681 (PMC11082093; doi:10.1002/ctm2.1681)

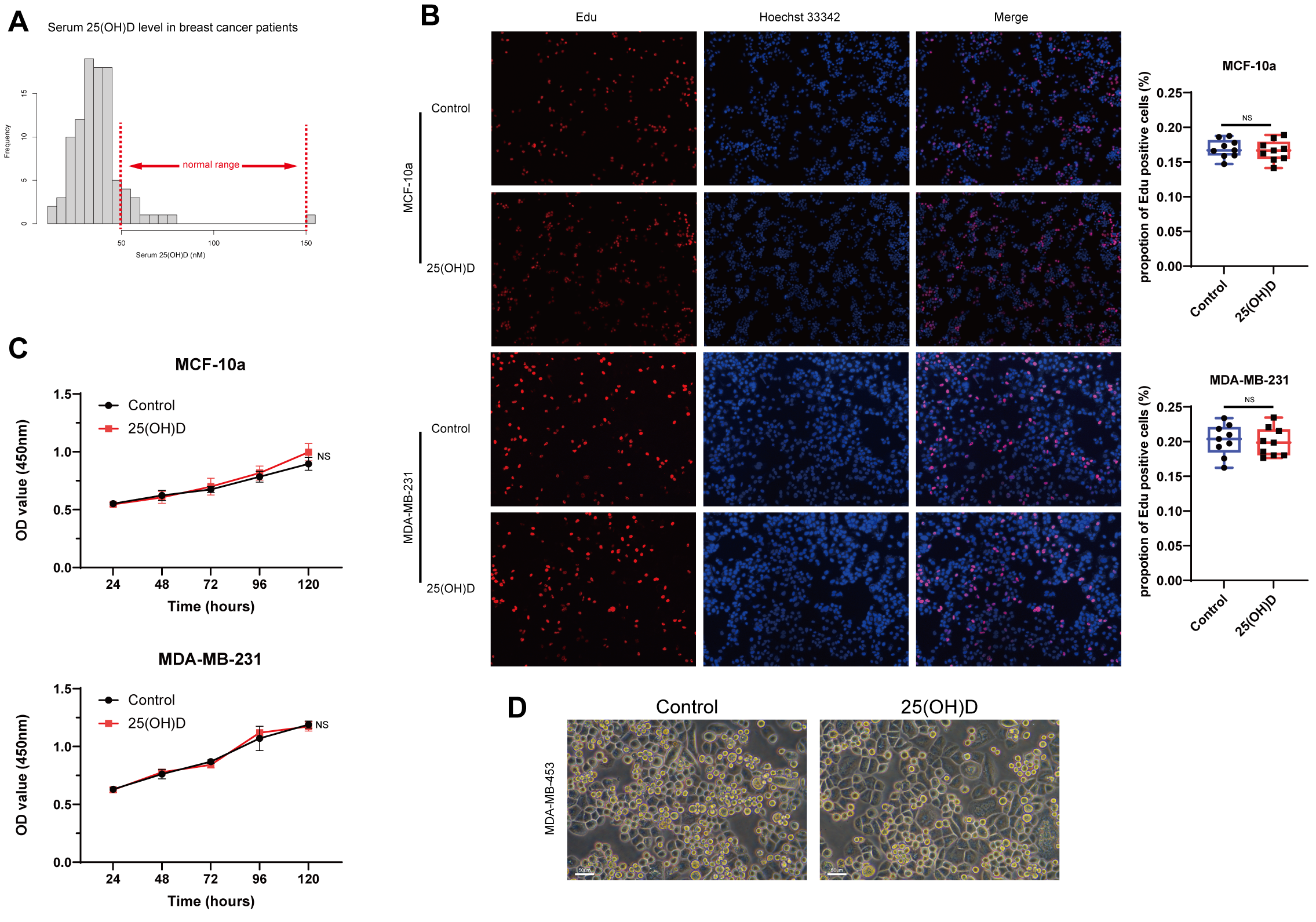

Supplement: Supplementary file 1 — Supporting Information [file CTM2-14-e1681-s007.tif]

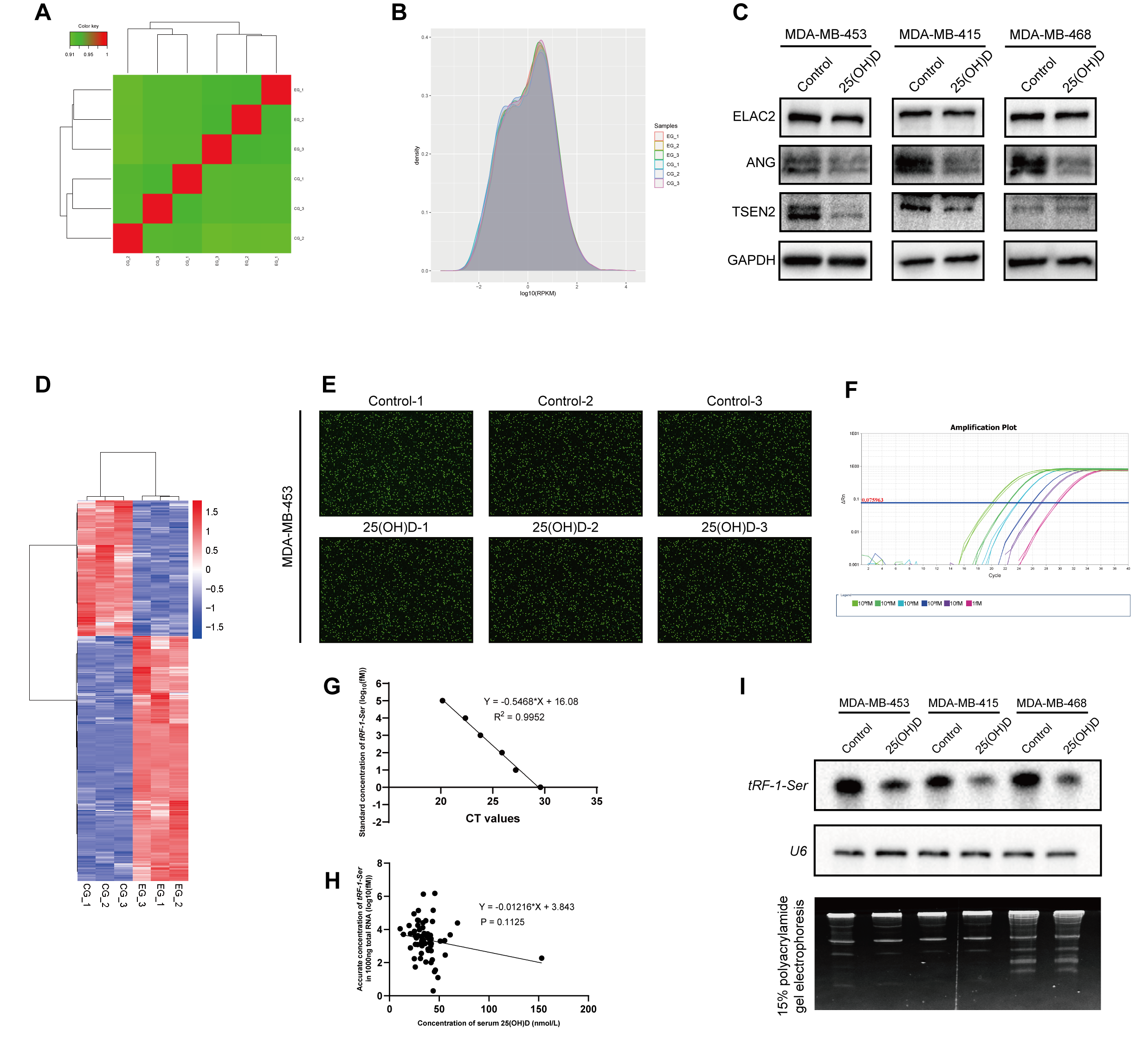

Supplement: Supplementary file 2 — Supporting Information [file CTM2-14-e1681-s006.tif]

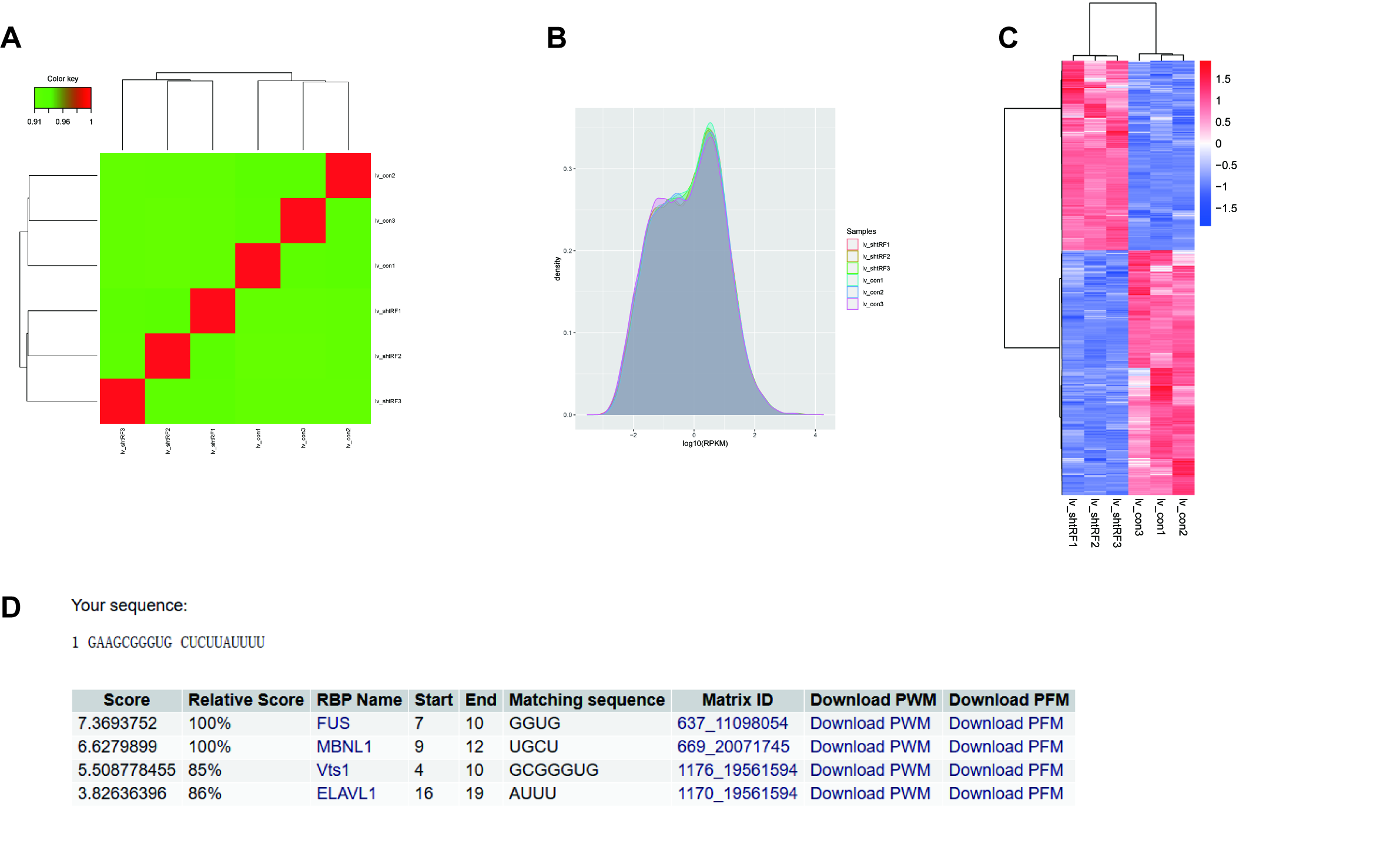

Supplement: Supplementary file 3 — Supporting Information [file CTM2-14-e1681-s002.tif]

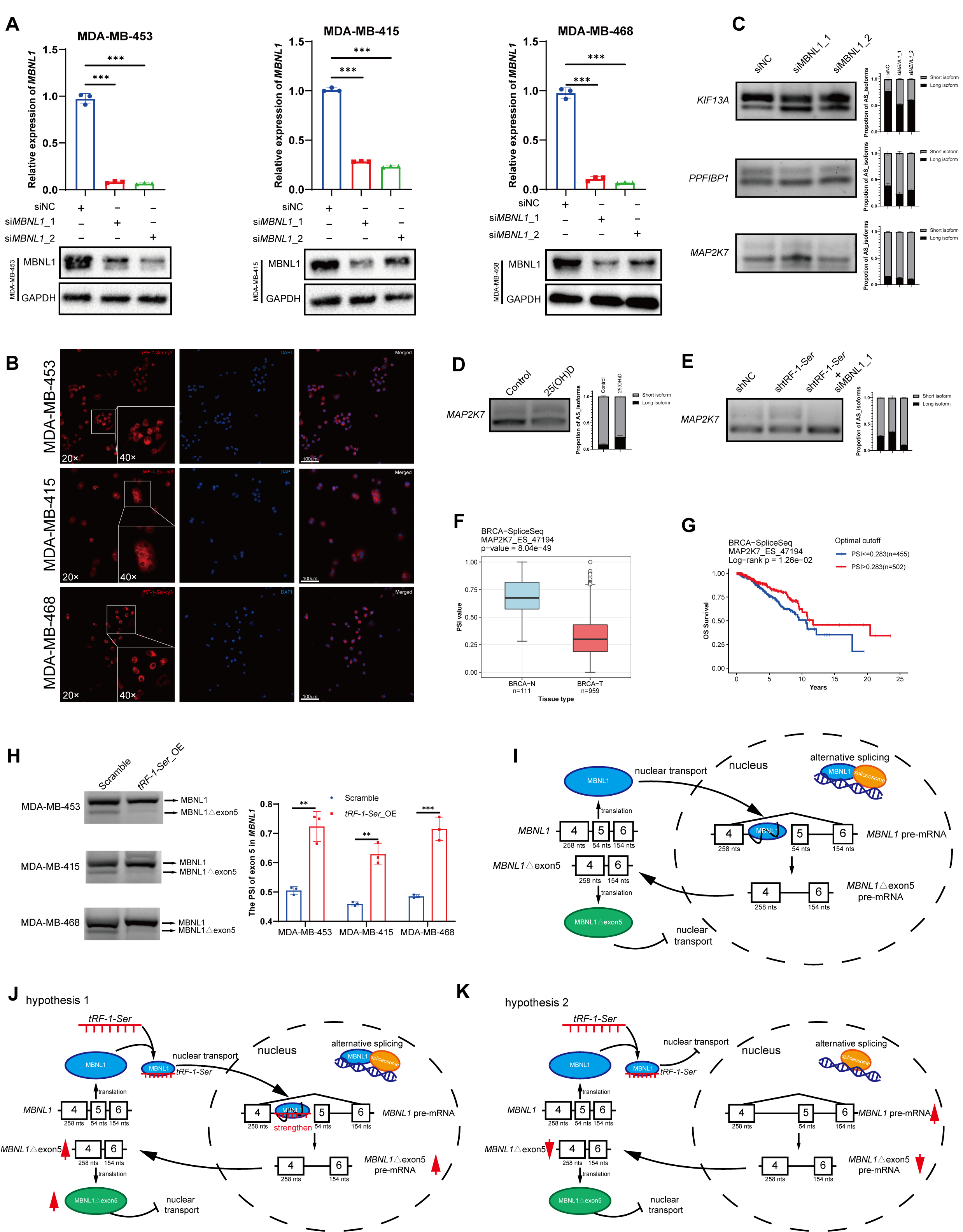

Supplement: Supplementary file 4 — Supporting Information [file CTM2-14-e1681-s009.tif]

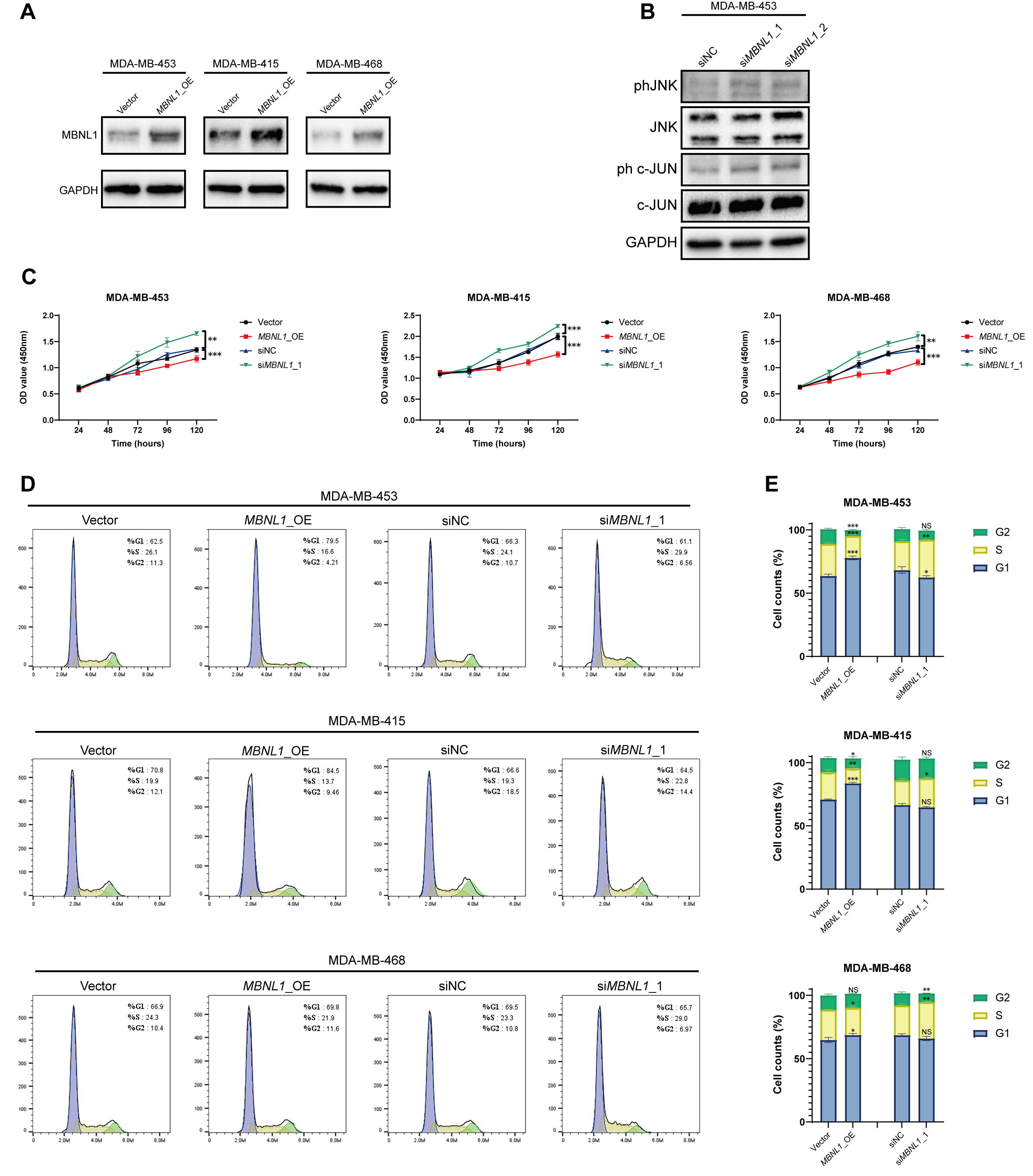

Supplement: Supplementary file 5 — Supporting Information [file CTM2-14-e1681-s004.tif]

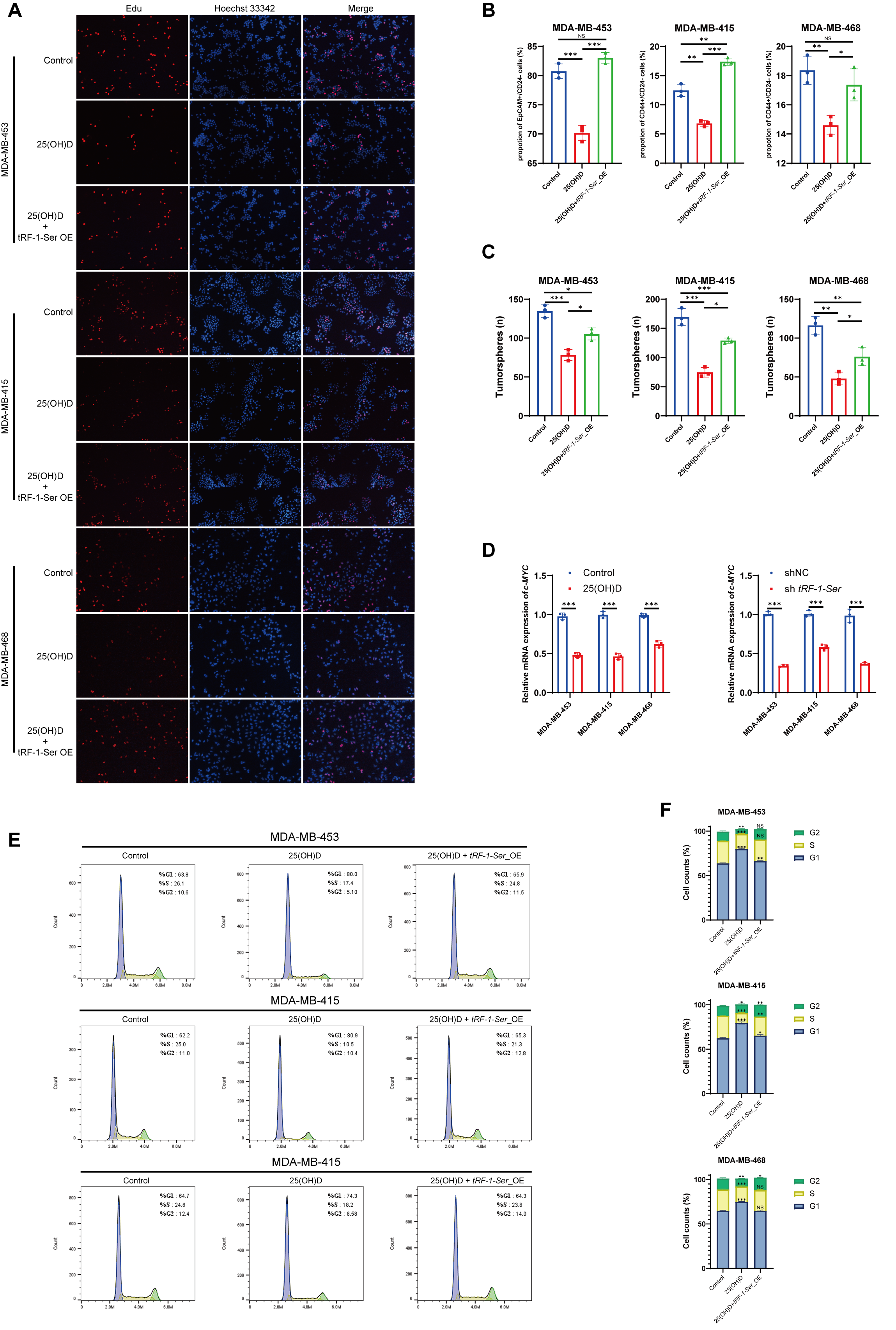

Supplement: Supplementary file 6 — Supporting Information [file CTM2-14-e1681-s005.tif]

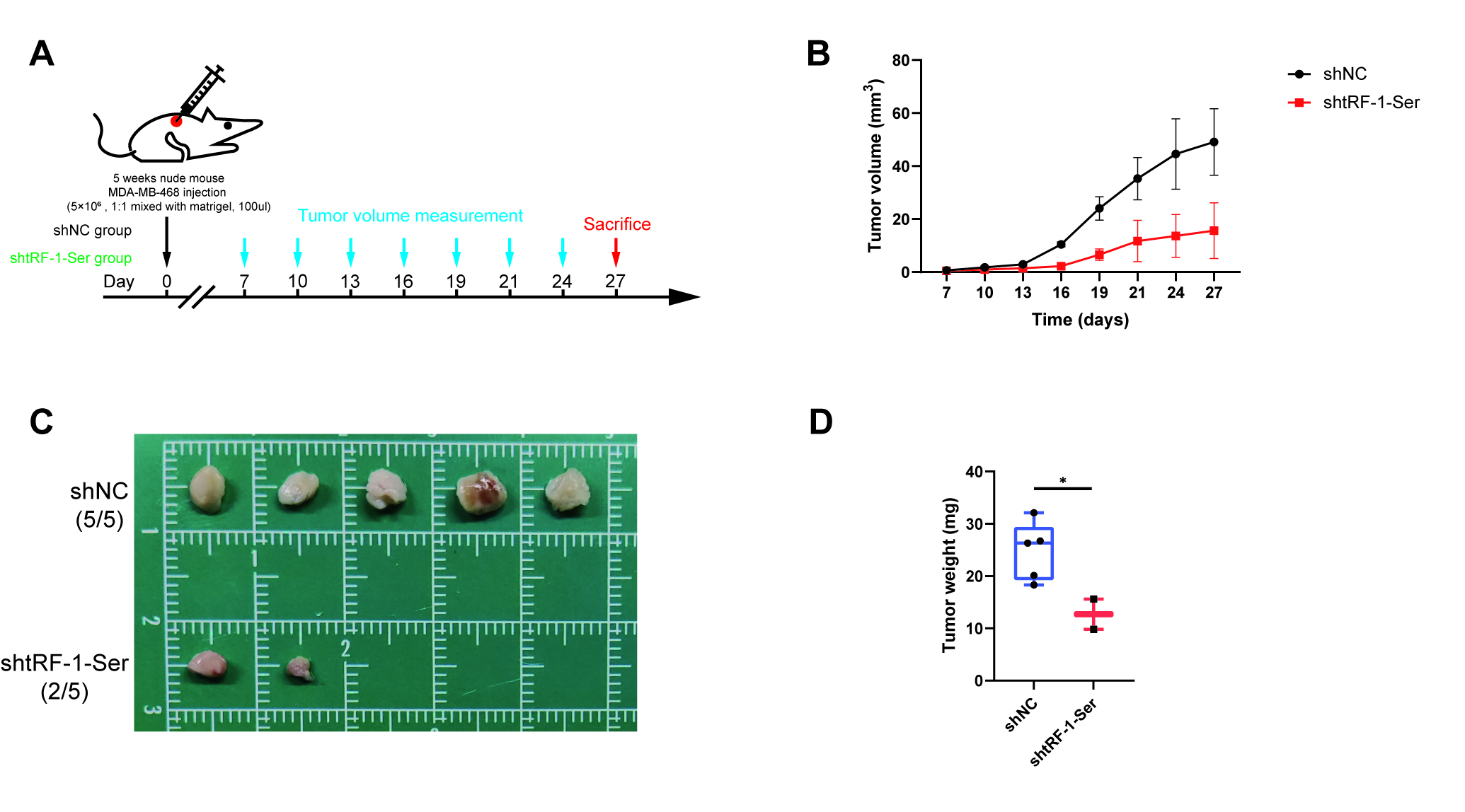

Supplement: Supplementary file 7 — Supporting Information [file CTM2-14-e1681-s010.tif]
